# Downregulation of exhausted cytotoxic T cells in gene expression networks of multisystem inflammatory syndrome in children

**DOI:** 10.1038/s41467-021-24981-1

**Published:** 2021-08-11

**Authors:** Noam D. Beckmann, Phillip H. Comella, Esther Cheng, Lauren Lepow, Aviva G. Beckmann, Scott R. Tyler, Konstantinos Mouskas, Nicole W. Simons, Gabriel E. Hoffman, Nancy J. Francoeur, Diane Marie Del Valle, Gurpawan Kang, Anh Do, Emily Moya, Lillian Wilkins, Jessica Le Berichel, Christie Chang, Robert Marvin, Sharlene Calorossi, Alona Lansky, Laura Walker, Nancy Yi, Alex Yu, Jonathan Chung, Matthew Hartnett, Melody Eaton, Sandra Hatem, Hajra Jamal, Alara Akyatan, Alexandra Tabachnikova, Lora E. Liharska, Liam Cotter, Brian Fennessy, Akhil Vaid, Guillermo Barturen, Hardik Shah, Ying-chih Wang, Shwetha Hara Sridhar, Juan Soto, Swaroop Bose, Kent Madrid, Ethan Ellis, Elyze Merzier, Konstantinos Vlachos, Nataly Fishman, Manying Tin, Melissa Smith, Hui Xie, Manishkumar Patel, Kai Nie, Kimberly Argueta, Jocelyn Harris, Neha Karekar, Craig Batchelor, Jose Lacunza, Mahlet Yishak, Kevin Tuballes, Ieisha Scott, Arvind Kumar, Suraj Jaladanki, Charuta Agashe, Ryan Thompson, Evan Clark, Bojan Losic, Lauren Peters, The Mount Sinai COVID-19 Biobank Team, Charuta Agashe, Priyal Agrawal, Eziwoma Alibo, Kelvin Alvarez, Angelo Amabile, Steven Ascolillo, Rasheed Bailey, Priya Begani, Cansu Cimen Bozkus, Paloma Bravo, Stacey-Ann Brown, Mark Buckup, Larissa Burka, Lena Cambron, Gina Carrara, Serena Chang, Steven T. Chen, Jonathan Chien, Mashkura Chowdhury, Dana Cosgrove, Francesca Cossarini, Arpit Dave, Travis Dawson, Bheesham Dayal, Maxime Dhainaut, Rebecca Dornfeld, Katie Dul, Nissan Eber, Frank Fabris, Jeremiah Faith, Dominique Falci, Susie Feng, Marie Fernandes, Daniel Geanon, Joanna Grabowska, Gavin Gyimesi, Maha Hamdani, Diana Handler, Manon Herbinet, Elva Herrera, Arielle Hochman, Jaime Hook, Laila Horta, Etienne Humblin, Jessica S. Johnson, Subha Karim, Geoffrey Kelly, Jong Kim, Dannielle Lebovitch, Brian Lee, Grace Lee, Gyu Ho Lee, Jacky Lee, John Leech, Michael B. Leventhal, Katherine Lindblad, Alexandra Livanos, Rosalie Machado, Zafar Mahmood, Kelcey Mar, Glenn Martin, Shrisha Maskey, Paul Matthews, Katherine Meckel, Saurabh Mehandru, Cynthia Mercedes, Dara Meyer, Gurkan Mollaoglu, Sarah Morris, Marjorie Nisenholtz, Merouane Ounadjela, Vishwendra Patel, Cassandra Pruitt, Shivani Rathi, Jamie Redes, Ivan Reyes-Torres, Alcina Rodrigues, Alfonso Rodriguez, Vladimir Roudko, Evelyn Ruiz, Pearl Scalzo, Alessandra Soares Schanoski, Pedro Silva, Hiyab Stefanos, Meghan Straw, Collin Teague, Bhaskar Upadhyaya, Verena Van Der Heide, Natalie Vaninov, Daniel Wacker, Hadley Walsh, C. Matthias Wilk, Jessica Wilson, Li Xue, Naa-akomaah Yeboah, Sabina Young, Nina Zaks, Renyuan Zha, Panagiotis Roussos, Jun Zhu, Wenhui Wang, Andrew Kasarskis, Benjamin S. Glicksberg, Girish Nadkarni, Dusan Bogunovic, Cordelia Elaiho, Sandeep Gangadharan, George Ofori-Amanfo, Kasey Alesso-Carra, Kenan Onel, Karen M. Wilson, Carmen Argmann, Supinda Bunyavanich, Marta E. Alarcón-Riquelme, Thomas U. Marron, Adeeb Rahman, Seunghee Kim-Schulze, Sacha Gnjatic, Bruce D. Gelb, Miriam Merad, Robert Sebra, Eric E. Schadt, Alexander W. Charney

**Affiliations:** 1 Department of Genetics and Genomic Sciences, Icahn School of Medicine at Mount Sinai, New York, NY USA; 2 Icahn Institute of Data Science and Genomics Technology, New York, NY USA; 3 Graduate School of Biomedical Sciences, Icahn School of Medicine at Mount Sinai, New York, NY USA; 4 Department of Psychiatry, Icahn School of Medicine at Mount Sinai, New York, NY USA; 5 Tisch Cancer Institute, Icahn School of Medicine at Mount Sinai, New York, NY USA; 6 Department of Medicine, Division of Surgery, Icahn School of Medicine at Mount Sinai, New York, NY USA; 7 Precision Immunology Institute, Icahn School of Medicine at Mount Sinai, New York, NY USA; 8 Icahn School of Medicine at Mount Sinai, New York, NY USA; 9 Human Immune Monitoring Center, Icahn School of Medicine at Mount Sinai, New York, NY USA; 10 Department of of Rehabilitation and Human Performance, Icahn School of Medicine at Mount Sinai, New York, NY USA; 11 Department of Medical Genomics, Center for Genomics and Oncological Research Pfizer/University of Granada/Andalusian Regional Government (GENYO), Granada, Spain; 12 Mount Sinai COVID Informatics Center, New York, NY USA; 13 Department of Medicine, Mount Sinai, New York, NY USA; 14 Hasso Plattner Institute for Digital Health at Mount Sinai, New York, NY USA; 15 Charles Bronfman Institute for Personalized Medicine, New York, NY USA; 16 Department of Urology, Icahn School of Medicine at Mount Sinai, New York, NY USA; 17 Departments of Pediatrics, Icahn School of Medicine at Mount Sinai, New York, NY USA; 18 Department of Oncological Sciences, Icahn School of Medicine at Mount Sinai, New York, NY USA; 19 Division of Hematology and Oncology, Icahn School of Medicine at Mount Sinai, New York, NY USA; 20 Department of Pathology, Icahn School of Medicine at Mount Sinai, New York, NY USA; 21 Mindich Child Health and Development Institute at Mount Sinai, New York, NY USA; 22 Black Family Stem Cell Institute, New York, NY USA; 23 Sema4, a Mount Sinai Venture, Stamford, CT USA

**Keywords:** Antimicrobial responses, Inflammation, Systems analysis, Prognostic markers

## Abstract

Multisystem inflammatory syndrome in children (MIS-C) presents with fever, inflammation and pathology of multiple organs in individuals under 21 years of age in the weeks following severe acute respiratory syndrome coronavirus 2 (SARS-CoV-2) infection. Although an autoimmune pathogenesis has been proposed, the genes, pathways and cell types causal to this new disease remain unknown. Here we perform RNA sequencing of blood from patients with MIS-C and controls to find disease-associated genes clustered in a co-expression module annotated to CD56^dim^CD57^+^ natural killer (NK) cells and exhausted CD8^+^ T cells. A similar transcriptome signature is replicated in an independent cohort of Kawasaki disease (KD), the related condition after which MIS-C was initially named. Probing a probabilistic causal network previously constructed from over 1,000 blood transcriptomes both validates the structure of this module and reveals nine key regulators, including TBX21, a central coordinator of exhausted CD8^+^ T cell differentiation. Together, this unbiased, transcriptome-wide survey implicates downregulation of NK cells and cytotoxic T cell exhaustion in the pathogenesis of MIS-C.

## Abstract (web summary)

Multisystem inflammatory syndrome in children (MIS-C) onsets in COVID-19 patients with manifestations similar to Kawasaki disease (KD). Here the author probe the peripheral blood transcriptome of MIS-C patients to find signatures related to natural killer (NK) cell activation and CD8+ T cell exhaustion that are shared with KD patients.

## Introduction

Multisystem inflammatory syndrome in children (MIS-C) presents with fever, inflammation, and pathology of multiple organs in individuals under 21 years of age in the weeks following severe acute respiratory syndrome coronavirus 2 (SARS-CoV-2) infection^1–3^. MIS-C was initially named Kawasaki-like disease due to perceived clinical presentation similarities between the two diseases^4^. Kawasaki disease (KD) is an acute febrile illness that presents with fever, rash, swelling of the hands, feet, and lymph nodes, and irritation and inflammation of the eyes, mouth, lips, and throat in children younger than 5 years of age^5^. Both MIS-C and KD have similar clinical presentations but unknown molecular causes. Here we show the first transcriptome-wide survey of MIS-C, implicating NK cells and exhausted CD8^﻿+^ T cells as downregulated in MIS-C.

To explore the molecular landscape of MIS-C, we establish here a differential expression (DE) gene signature of disease and utilize co-expression and Bayesian probabilistic-causal networks to analyze RNA-sequencing (RNA-seq) data from MIS-C patients. Co-expression networks group genes into functional modules that can give greater biological context to the mathematically driven DE gene signatures. Bayesian probabilistic-causal networks (BN) build directed-acyclic graphs (DAGs) from the expression data that can be used to understand how genes interact with each other. Key driver analysis (KDA) is used on the BN to establish genes that are key regulators of the network, defined as either global key regulators (genes that are highly connected within the network) or local key regulators (genes that are significantly upstream of specific genes of interest).

Our approach reveals aberrant NK and CD8^+^ T cell biology in MIS-C patients. These cytotoxic cells can regulate one another in response to viral infection^6^, and the depletion of NK cells has been shown to disrupt CD8^+^ T cell exhaustion, a process where virus-specific CD8^+^ T cells have poor cytokine output, cytolytic activity, and impaired proliferative capacity are actively inhibited^7,8^. Disruption in CD8^+^ T cell exhaustion can lead to severe and even fatal T cell immunopathology after viral infection^7,8^ whereas its presence can improve inflammatory disease symptoms^9^.

Altogether, this network-based approach implicates a decrease in NK cells, CD8^+^ T cells, and a downregulated module of genes annotated to mature exhausted CD8^+^ T (Tex) cells and CD56^dim^CD57^+^ NK cells in MIS-C. This decrease in NK cells may lead to a lack of CD8^+^ T cell exhaustion which has been shown to lead to severe inflammatory disease that may have similarities to MIS-C. This finding replicates in an independent cohort of KD cases, suggesting common molecular disease etiologies for MIS-C and KD.

## Results

**Fig. 1:**
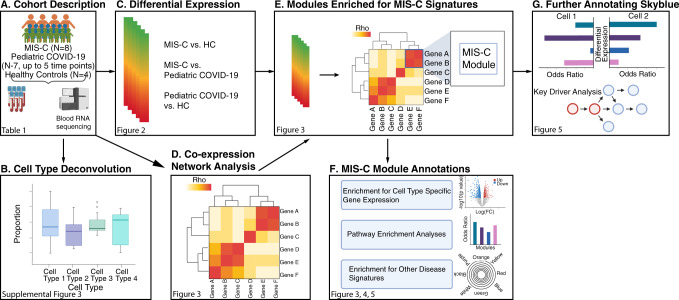
Study overview. Workflow of the analyses presented in the paper. **A** RNA-seq was generated on whole blood samples from MIS-C cases, pediatric COVID-19 cases, and healthy controls. **B** Deconvolution estimated the relative cell-type proportions in each transcriptome, which were compared between cases and controls to identify the immune cell types involved in the pathogenesis of MIS-C. **C** Expression of each gene in the transcriptome was tested for association with disease, resulting in a MIS-C signature that was queried to resolve the dysregulated molecular pathways. **D** Co-expression network construction organized genes into coherent units called modules. **E** and **F** Modules loaded with genes of the MIS-C signature were empirically identified (**E**), validated using DE signatures from a large Kawasaki disease (KD) gene-expression dataset (**F**), and functionally annotated to pathway, cell type, and other disease signatures (**F**). **G** The module with the strongest enrichment for MIS-C that also enriched for KD signatures was further annotated to pinpoint cell subtypes, and key regulators of the processes captured by this module were identified in a regulatory network built from whole blood gene expression in an independent cohort. This figure was created with BioRender.com.

RNA-seq was performed on 30 whole-blood specimens from MIS-C cases (eight specimens from eight individuals), pediatric COVID-19 cases (18 specimens from seven individuals), and healthy controls (HCs; four specimens from four individuals) collected as part of their clinical visits (Fig. 1a, Table 1, Supplementary Data 1–3, see the “Methods” section). For MIS-C patients, all visits were at the acute disease stage (requiring an emergency visit to the hospital). Quality control procedures identified eight covariates driving variance in gene expression, which were accounted for in subsequent analyses (Supplementary Fig. 1a–d). One of these covariates was age, which was mismatched between cases and controls due to the circumstances of the current pandemic. It was modeled by deriving an estimate of the transcriptional age of each study participant (see the “Methods” section). This approach allowed us to account for the effect of age on gene expression without removing the effects of disease, as would happen if age itself were included as a covariate in our analyses (Supplementary Fig. 1b–d, Supplementary Fig. 2, and Supplementary Data 4).

**Table 1:** Sample description.

Index	Age	Gender	Ethnicity	Comorbidities	Immunosuppressive
*MIS-C*					
M1	0–5	Male	Hispanic	None	None
M2	6–11	Female	Hispanic	None	None
M3	12–17	Male	Black	Asthma	None
M4	0–5	Female	Hispanic	None	None
M5	18–23	Male	Hispanic	Asthma	None
M6	12–17	Male	Hispanic	Appendicitis	None
M7	12–17	Female	Black	MDD, PTSD	None
M8	6–11	Female	Hispanic	None	None
*Pediatric COVID-19*					
C1	6–11	Female	Hispanic	B-cell ALL	Chemotherapy
C2	18–23	Male	Hispanic	Asthma	None
C3	0–5	Male	Hispanic	Autoimmune Anemia	Steroids
C4	18–23	Female	Not Reported	Pineoblastoma	Chemotherapy
C5	18–23	Male	Hispanic	B-cell ALL	Chemotherapy
C6	6–11	Male	White	Gastrochisis	Tacrolimus
C7	12–17	Male	Black	IBD	Infliximab
*Healthy controls*					
H1	24–29	Female	Asian	None	None
H2	35–40	Male	White	None	None
H3	24–29	Female	White	None	None
H4	35–40	Female	White	None	None

Demographic and clinical variables for the MIS-C cases (*N* = 8), pediatric COVID-19 cases (*N* = 7), and healthy controls (*N* = 4). Index column refers to the individual identifiers. Age is presented in years. Sex, ethnicity, and comorbidities are as reported in the electronic medical record. Immunosuppressants indicate the usage of chronic immunosuppressive therapies for any indication. Further clinical data can be found in Supplementary Data 1–3.

*MDD* major depressive disorder, *PTSD* post-traumatic stress disorder, *ALL* acute lymphoblastic leukemia, *IBD* inflammatory bowel disease.

**Fig. 2:**
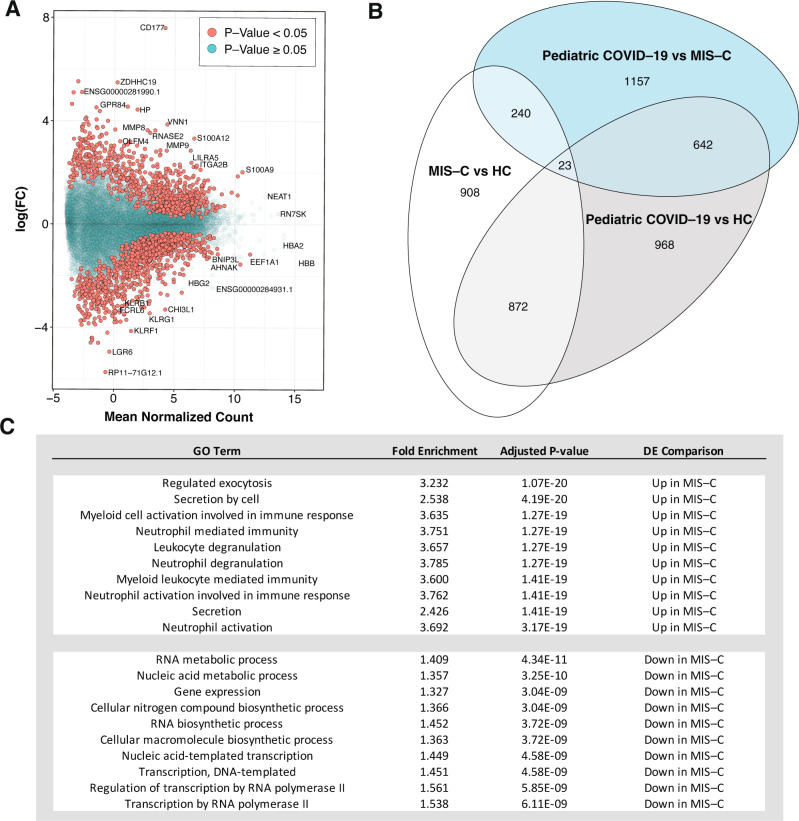
Differential expression analyses identify the transcriptional signature of MIS-C. **A** Differential expression (DE) analysis for MIS-C patients versus HCs. The *x*-axis is the mean normalized count for each gene and the *y*-axis is the log_2_(fold-change) for the differential expression. Positive and negative log_2_(fold change) represent genes upregulated and downregulated in MIS-C, respectively, and the significance of association between gene expression and MIS-C status is indicated by the color of the dots as defined in the legend. **B** Overlap of MIS-C and pediatric COVID-19 transcriptional signatures: Venn diagram of the overlap of genes across DE signatures. Each comparison is labeled on the plot. **C** GO terms for MIS-C signature: GO term enrichment results for the top 10 upregulated and downregulated processes in MIS-C compared to HCs. Two-sided Fisher tests were used and *p*-values were adjusted for multi-testing as described in the “Methods” section. Full DE results and pathway enrichments for all comparisons in **B** can be found in Supplementary Data 7 and 8.

A flowchart of the analyses performed for this study is presented in Fig. 1. Transcriptional differences between the three groups in our cohort were evaluated by cell type deconvolution and DE analyses. Deconvolution identified cytotoxic T cells as significantly downregulated in MIS-C compared to HCs, and B-cells as significantly downregulated in pediatric COVID-19 cases compared to MIS-C cases and upregulated in pediatric COVID-19 cases compared to HCs (Supplementary Fig. 3, Supplementary Data 5 and 6*,* see the “Methods” section). As expected at this sample size, DE did not reveal any differentially expressed genes (DEGs) between MIS-C and HCs. However, using the π_1_ statistic, which provides a lower bound of the proportion of genes tested that truly deviate from the null hypothesis, we estimated that ~15% of genes tested between MIS-C and HCs were true DEGs^10–12^. The MIS-C signature was therefore defined as the 2043 DEGs with a *p*-value < 0.05 (Fig. 2, Supplementary Data 7). In the same way, we defined the pediatric COVID-19 signature compared to HCs (2505 DEGs) and the signature differentiating MIS-C from pediatric COVID-19 (2062 DEGs) (Figs. 1c, 2b, and Supplementary Data 7). To identify known biological processes associated with MIS-C and pediatric COVID-19, we tested these three upregulated and downregulated DE signatures for the enrichment of canonical pathways (Fig. 2c and Supplementary Data 8). The top gene ontology (GO) terms associated with the upregulated MIS-C signature were related to the immune system, while the top GO terms associated with the downregulated MIS-C signature were related to the regulation of gene expression. The latter was also observed for the downregulated pediatric COVID-19 signature. The upregulated pediatric COVID-19 signature was most strongly enriched for the GO term ‘vesicle-mediated transport’ (odds ratio, OR = 1.61, FDR = 2.17E−09). Comparing MIS-C to pediatric COVID-19, genes upregulated in MIS-C were enriched for multiple immune systems GO terms, and genes upregulated in pediatric COVID-19 were most strongly enriched for the GO term ‘cellular macromolecule catabolic process’ (OR = 1.88, FDR = 3.79E−11).

**Fig. 3:**
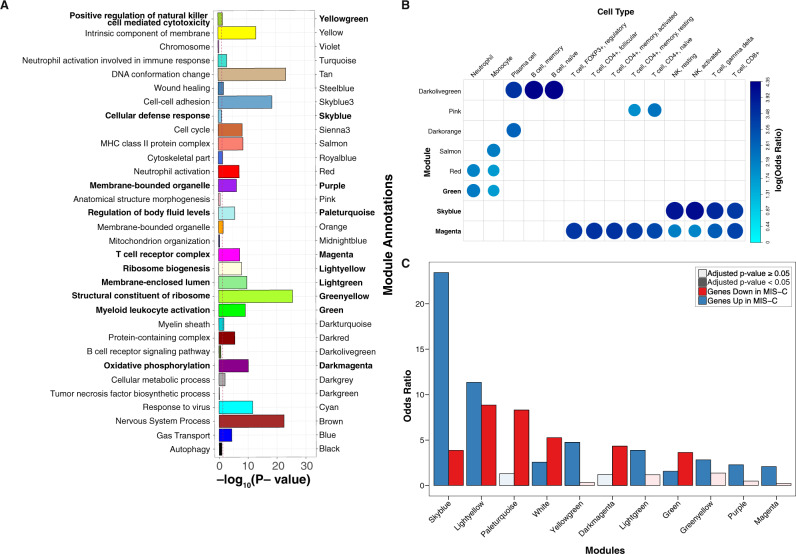
Co-expression network analysis identifies modules of genes dysregulated in MIS-C. **A** Module GO term enrichments. The *y*-axis is the most significant GO term associated with each module, the *x*-axis is the −log_10_(adjusted *p*-value) for the enrichment (performed using Fisher’s test). Bars are colored by module names, which are also specified for clarity. Only modules with an enrichment *p*-value < 1 are shown. The threshold for significance, −log_10_(0.05), is indicated by the red dashed line. In bold lettering are the modules enriched for the MIS-C signature. **B** Module cell type signature enrichments. The *x*-axis and *y*-axis are the names of the modules and of the cell type signatures, respectively. All signatures shown here are derived from the LM22 reference (Newman et al. *Nat. Methods*, 2015). The color and size of the circles represents the log_2_(odds ratio) of the enrichments as defined in the legend. In bold are the modules enriched for the MIS-C signature. Only enriched cell types are shown. **C** Modules enrichment for MIS-C signatures. The *x*-axis is the module names and the *y*-axis the odds ratio of the enrichment of the modules for the genes upregulated and downregulated in MIS-C. Only modules significantly enriched for MIS-C DEGs are shown. The color of the bars represent direction and the opacity represent the significance as defined in the legend. All *p*-values were adjusted for multi-testing as described in the “Methods” section. All module enrichments can be found in Supplementary Data 7, 9–12, 21.

To dissect the MIS-C signature we built a co-expression network that organized the transcriptome into 37 non-overlapping functional groups of genes called modules (Fig. 1d)^13^. Modules were functionally annotated by testing their overlap with canonical pathways and cell type-specific expression signatures (Fig. 1f), revealing a biologically coherent network structure that formed the basis for much of the remaining analyses (Fig. 3, Supplementary Data 9–12, and Supplementary Fig. 4)^12,14^. Projecting DEGs onto this network (Fig. 1e) identified 11 modules enriched for the MIS-C signature, with six modules enriched for upregulated DEGs, nine for downregulated DEGs, and four for both (Fig. 3c). The module most significantly impacted in MIS-C was skyblue (159 genes; Fisher’s exact test odds ratio [OR] for downregulated MIS-C DEGs = 23.44, adjusted *p*-value = 4.33E−66). This module was annotated to CD8^+^ T cells and NK cells, the cornerstones of cytotoxic immunity, corroborating the observations from cell type deconvolution described above (Fig. 3b, Supplementary Fig. 4, and Supplementary Data 10–12; see the “Methods” section). To ensure that the skyblue enrichment was not driven by the age mismatch between MIS-C and HCs, we repeated these analyses utilizing age-matched controls sequenced for an earlier study^15^ (Supplementary Data 13 and 14) and found consistent enrichment of skyblue for genes downregulated in MIS-C (Supplementary Data 10–12).

**Fig. 4:**
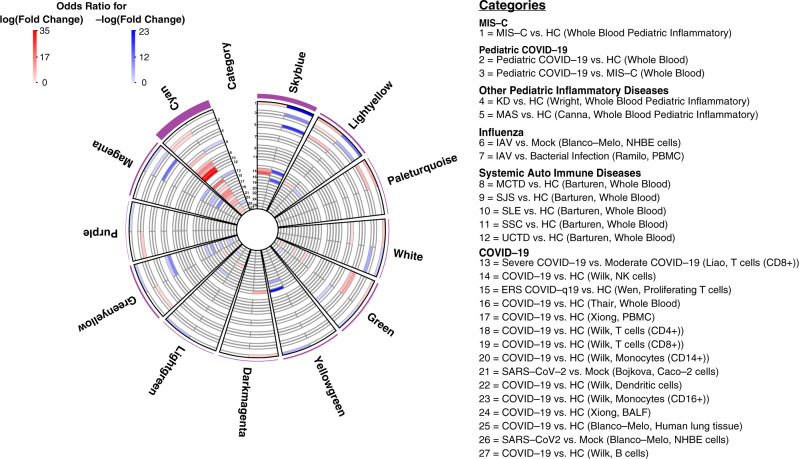
Disease enrichments in MIS-C modules and cyan. Slices are modules and each circular row represents the corresponding disease signature defined in the legend on the right. The purple outer rim of the plot represents the sum of the OR for all enrichments in that slice (performed using Fisher’s test). Each slice is divided into two components that show the OR for the enrichment of genes with ±log(fold change) in the corresponding disease signature (red and blue, respectively, as defined in legend). Numbers in the category slice map the circular rows to disease signatures. ORs are shown for disease signature enrichment adjusted *p*-values < 0.05. Diseases were grouped in biologically meaningful clusters as defined in the legend. Parenthesis in the legend refers to the source of the signature and the tissue or cell type assessed. References for disease signatures are defined in the “Methods” section and all enrichments can be found in Supplementary Data 15.

We sought to replicate the association of these 11 modules to MIS-C in an independent cohort. Due to the recent emergence and rarity of MIS-C at the time of writing, such a cohort could not be acquired. Instead, we leveraged the similarity between MIS-C and KD^4^, deriving a DE signature for the latter by re-analyzing an independent cohort of 78 KD cases and 55 controls with blood gene-expression data^16^. As with MIS-C, skyblue was the module most strongly impacted in KD (Fisher’s exact test OR for downregulated KD genes = 19.18, adjusted *p*-value = 1.64E−04), supporting the central role of these genes in MIS-C (Fig. 4 and Supplementary Data 15). Three other modules were also significantly enriched in the same direction for both MIS-C and KD (Fig. 4 and Supplementary Data 15). To rule out non-specific pediatric inflammatory states as the explanation for the observed overlap, we also projected DE signatures from macrophage activation syndrome (MAS) and neonatal-onset multisystem inflammatory disease (NOMID) onto the MIS-C modules^17^. Minimal overlap was seen between MIS-C and these diseases (Fig. 4), supporting the KD case-control cohort as a suitable means for replicating MIS-C findings given the absence of independent MIS-C case-control cohorts (Fig. 4).

We next harnessed the co-expression network structure to determine the molecular mechanisms linking SARS-CoV-2 infection to MIS-C. Eight of the 11 MIS-C modules were enriched in the same direction in at least one of a comprehensive set of 23 COVID-19 signatures either generated for this study or curated from independent studies that used a wide range of molecular profiling approaches (Fig. 1e–f, Fig. 4 and Supplementary Data 15; see the “Methods” section). To assess whether this overlap was due to the SARS-CoV-2 virus or viral infection more broadly, we projected DE signatures for influenza A virus (IAV) and Ebola virus disease (EVD) onto our network. EVD was not enriched in any MIS-C modules, and while IAV was enriched in a subset, none of these modules were shared between MIS-C and pediatric COVID-19 (Fig. 4 and Supplementary Data 15), supporting the notion that the SARS-CoV-2 infection underlies the molecular link we identified between MIS-C and COVID-19. Notably absent from the eight modules these conditions shared was the cyan module, which showed no enrichment for MIS-C but was found enriched for 61% of the upregulated COVID-19 signatures tested (Fig. 4). This module was also enriched for IAV signatures (Fig. 4 and Supplementary Data 15). It was annotated to the ‘response to the virus’ GO term (OR = 5.22, FDR = 2.38E−12) and ‘interferon-stimulated CD4^+^ T cells’ (Fisher’s exact test OR = 47.31, adjusted *p*-value = 1.24E−39; Supplementary Data 10). The striking association of cyan to COVID-19 substantiates the hypothesis that imbalanced interferon response is a hallmark of COVID-19^18^, with a blunted response during the incubation phase of SARS-CoV-2 infection^19^ followed by over-compensation during the acute illness.

We performed several analyses to gain a deeper understanding of how skyblue and other modules confer risk for MIS-C. First, in light of recent suggestions that the pathophysiology of MIS-C overlaps that of classic autoimmune disease^20–22^, we assessed if MIS-C modules were enriched for transcriptional signatures of autoimmunity^23^. Neither skyblue nor the other 10 MIS-C modules were enriched for any of the seven autoimmune signatures tested using data from the most comprehensive transcriptome-wide study of autoimmunity to date (Fig. 4 and Supplementary Data 15), suggesting that if MIS-C is indeed an autoimmune reaction it is through other mechanisms. For most of the autoimmune signatures tested, upregulated genes were enriched in the cyan module that was strongly linked to COVID-19 (Fig. 4 and Supplementary Data 15).

**Fig. 5:**
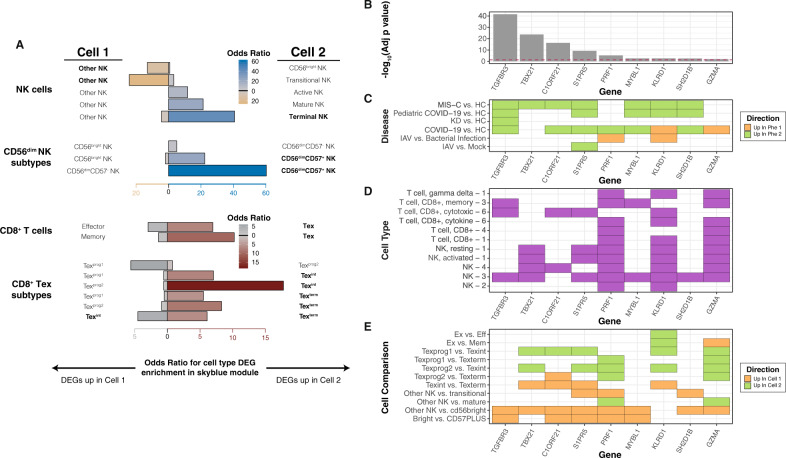
Further dissection of skyblue implicates cytotoxic lymphocyte subtypes in MIS-C. **A** Defining NK cell and CD8^+^ T cell subtypes in skyblue (Supplementary Data 19). The *x*-axis represents the OR for the enrichment of skyblue for specific cytotoxic cell subtypes and the *y*-axis the signatures used. Cell one and cell two refer to the cell subtypes being compared to generate the DE signatures projected onto skyblue and the direction of the bar is shown towards the upregulated cell type in the comparison. The color and length of bars represent the OR of the enrichment test as defined by the *x*-axis and the legends. For NK cells, we used signatures from Yang et al., *Nat. Commun.*, 2019 (labeled NK cells), and from Collins et al. *Cell* 2019 (labeled CD56^dim^ NK subtypes). For CD8^+^ T cells, we used signatures from Wherry, et al. *Immunity*, 2007 (labeled CD8^+^ T cells), and from Beltra et al. *Immunity*, 2020 (labeled CD8^+^ Tex subtypes). Detailed descriptions of the subtypes can be found in the Methods. **B**–**E** Skyblue key drivers measures. The x-axes of the panels represent the key driver genes. **B** Key driver analysis results (hypergeometric test).The *y*-axis of this panel is the −log_10_(Bonferroni adjusted *p*-value) of key driver analysis (Supplementary Data 21). The red dashed line is the significance threshold at −log_10_(0.05). **C** Related disease differential expression. In this panel, the *y*-axis is the disease name corresponding to Fig. 4. The color represents the direction in the comparison as defined in the legend (defined per the associated reference). **D** Cell-type-specific expression of key driver genes (Supplementary Data 22). The *y*-axis is the cell type signatures from the following references: 1 = Newman et al. Nature Methods, 2015; 2 = Park et al. Science, 2020; 3 = Wilk et al. Nature Medicine, 2020; 4 = Liao et al. Nature Medicine, 2020; and 6 = Szabo et al. Nature Communications, 2019. **E** CD8^+^ T cell and NK cell subtype-specific signatures. The *y*-axis is the cell-type name corresponding to **A** and the color represents the direction in the comparison as defined in the legend (defined per the associated reference).

Second, we sought to identify the subtypes of CD8^+^ T cells and NK cells in MIS-C by projecting subtype-specific cell signatures onto skyblue (Fig. 1g). Functional classes of CD8^+^ T cells that emerge following viral infection include effector, memory, and exhausted subtypes (Teff, Tmem, and Tex, respectively)^24–26^. Skyblue was enriched for DEGs upregulated in Tex compared to both Tmem (Fisher’s exact test OR = 10.23, adjusted *p*-value = 7.11E−4) and Teff (Fisher’s exact test OR = 6.96, adjusted *p*-value = 0.03; Fig. 5a and Supplementary Data 16 and 17)^25^. This was not observed for other MIS-C modules annotated to CD8^+^ T cells (Supplementary Fig. 5). Four transcriptional stages of Tex differentiation have been described^27^: two progenitor states (Tex^prog1^ and Tex^prog2^), an intermediate state (Tex^int^), and a terminally exhausted state (Tex^term^). Skyblue was found enriched for the signatures of mature stages (Tex^int^ and Tex^term^) compared to the progenitor phases (Tex^prog1^ and Tex^prog2^) (Fig. 5a). NK cells in human circulation are of two types, CD56^bright^ and CD56^dim^, with the latter being more common, more cytotoxic^28^, and roughly evenly split into CD57^+^ and CD57^−^ subtypes^29^. Skyblue was enriched for genes upregulated in CD56^dim^ compared to CD56^bright^, and this was driven exclusively by the CD56^dim^CD57^+^ NK subtype (Fisher’s exact test OR = 22.27, adjusted *p*-value = 1.12 × 10^–16^; Fig. 5a and Supplementary Data 16 and 17).

We validated the downregulation of CD8^+^ exhausted T cells using CyTOF in individuals with MIS-C, showing a significantly lower proportion of PD-1 high CD8^+^ T cells out of the CD8^+^ T cell population as compared to controls (Supplementary Fig. 6a–c, Supplementary Data 18 and 19). While the downregulation of the CD16^high^CD57^+^ NK cells (a proxy for the CD56^dim^CD57^+^ component of NK cells) was not clearly downregulated in the CyTOF results when compared to other NK populations, NK cells were significantly downregulated overall (Supplementary Fig. 6a, b, d, Supplementary Data 18). This finding potentially implies that our result mapping skyblue to CD56^dim^CD57^+^ NK cells may not be directly representative of the relative abundance of this subtype of NK cells but may rather indicate alteration of their specific functions. Together these analyses validate the implication of exhausted CD8^+^ T cells and NK cells in MIS-C.

Third, probabilistic causal network modeling was leveraged to identify putative key regulators of MIS-C pathogenesis in skyblue (Fig. 1e, g). As our dataset was not sufficiently large for Bayesian network (BN) construction, we utilized a BN recently constructed from RNA-seq of 1032 blood specimens^30^ in which skyblue was strongly conserved, with a subnetwork more than 29-fold enriched for genes in skyblue (*p*-value = 1.5E−47). Key regulators were defined as genes that, when perturbed, are predicted to alter the regulatory state of a module (Supplementary Data 20)^31^. Nine key regulators of skyblue were found: *TGFBR3, TBX21, C1ORF21, S1PR5, PRF1, MYBL1, KLRD1, SH2D1B*, and *GZMA*. These key regulators were generally downregulated in MIS-C and other diseases, highly expressed in CD8^+^ T cells and NK cells, and differentially expressed in the mature stages of Tex differentiation and in CD56^dim^CD57^+^ compared to other NK cell subtypes (Fig. 5b–e). We hypothesize that these are important both for understanding the etiology of MIS-C and as potential therapeutic targets, therefore warranting further study.

In conclusion, here we present one of the first genome-wide investigations of MIS-C, implicating a decrease in NK cells, CD8^+^ T cells, and a downregulated module of genes annotated to mature Tex cells and CD56^dim^CD57^+^ NK cells. The predominant cytotoxic cells of the immune system, CD8^+^ T cells, and NK cells co-regulate one another in a myriad of ways^6^. NK cell-dependent exhaustion of effector CD8^+^ T cells has been described^7,8^, and, consistent with our findings, its absence can lead to severe and even fatal T cell immunopathology after viral infection^7,8^ whereas its presence can improve inflammatory disease symptoms^9^. The nine key regulators of skyblue all have known associations with NK cell and CD8^+^ T cell functions^32–41^ and a subset have been further associated with illnesses resembling MIS-C^42–47^. *TBX21* is the most promising therapeutic target candidate amongst these nine because it codes for T-bet, a biological switch in the transition of Tex^prog2^ to Tex^int^ during Tex differentiation^27^. Altogether, our study shows a scarcity of NK cells in individuals with MIS-C with a potential dysregulation of the remaining NK cells, including in their interactions with CD8^+^ T cells. This decrease in NK cell proportion accompanied by lack of CD8^+^ T cell exhaustion would create a sustained inflammatory environment that, in turn, could enhance autoreactivity, an emerging hallmark of MIS-C^20^. An important remaining question will be to uncover what propels the resolution of SARS-CoV-2 infection to lead to MIS-C in some cases.

## Methods

### Ethics

For all study participants, informed consent was obtained from either the participant or a legally authorized representative, and all study-related activities were conducted under the approval and oversight of the Icahn School of Medicine at Mount Sinai’s Institutional Review Board (IRB 20-03276).

### MIS-C cohort description

MIS-C cases (*N* = 8) ranged in age from 5 to 20 years old (mean = 11, standard deviation = 5.2), were largely free of other comorbidities, and balanced for gender. Past medical history included asthma (*N* = 2), mood and anxiety disorders (*N* = 1), and appendicitis (*N* = 1), but overall these were healthy and active children prior to developing MIS-C. They presented either by inter-hospital transfer or through the emergency room and were treated in the inpatient unit until stable for discharge home. All met the formal MIS-C diagnostic criteria as defined by the Centers for Disease Control^3^ and had positive SARS-CoV-2 serology at the time of sampling. Two patients also tested positive for SARS-CoV-2 PCR at the same time. On initial physical exam, the majority (*N* = 6) were found to have a fever and various combinations of the hallmark signs of KD: cervical lymphadenopathy, bilateral nonexudative conjunctivitis, and mucous membrane changes including cracked lips and strawberry tongue. Empiric broad-spectrum antibiotics were started in every patient upon admission due to concern for bacterial etiology. Cardiac enzymes drawn upon presentation were notable for significant elevations in brain natriuretic peptide (*N* = 5) and follow-up echocardiograms revealed reduced ejection fraction (*N* = 5), dilation or prominence of coronary arteries (*N* = 4), and mild mitral and/or tricuspid regurgitation (*N* = 4). All patients exhibited signs of cytokine release syndrome and thus were treated with tocilizumab, with most also receiving intravenous immunoglobulin and steroids before study sample collection. Hypotension or shock developed in five cases and three required vasopressors. Anticoagulation by enoxaparin was initiated in all patients to address the coagulopathy found on clinical laboratory tests.

All cases improved steadily over the course of days (average length of hospitalization was 5.9 days) and upon discharge, their symptoms had largely resolved and clinical laboratory metrics were trending in the right direction. At subsequent outpatient follow-up appointments, some cases reported fatigue, but by one-month post-discharge, all subjects were at their clinical baseline. They were maintained initially on low-molecular-weight heparin, then switched to baby aspirin until D-dimer returned to baseline. Repeat echocardiograms were reassuring that cardiac abnormalities were transient, demonstrating normal coronaries and ventricular function. Key demographic and clinical variables for all MIS-C participants are also provided in Table 1 and Supplementary Data 1–3.

### Pediatric COVID-19 cohort description

Pediatric COVID-19 cases (*N* = 7) were recruited by case referrals coming from treating physicians in the Mount Sinai Health System in New York City. They ranged in age between 5 months and 19 years old (mean age = 12.4, standard deviation = 7). Nearly all of the pediatric COVID-19 cases were chronically immunocompromised, with three suffering from comorbid malignancies requiring chemotherapy and three on immunosuppressive therapy for other indications. Only one case was not immunocompromised, this individual had a past medical history of asthma and an anxiety disorder. We provide a brief description of the clinical course of each pediatric COVID-19 case as it relates to the timing of study sample collection, using the individual indices in Table 1 and Supplementary Data 3, where can be found further key demographic and clinical variables for all pediatric COVID-19 participants.

C1 was a 6–11-year-old female with a past medical history of B-cell acute lymphoblastic leukemia followed closely in the oncology clinic who was admitted 12 days prior to the first study sample collection for febrile neutropenia in the setting of a positive SARS-CoV-2 PCR test. Over the course of 8 days in the hospital, she recovered with treatment for COVID-19 and was discharged in stable condition. She was sampled for the study at her subsequent outpatient chemotherapy visits, by which time she had recovered clinically from the acute COVID-19 illness. At the later study sample time points, the patient had both clinically recovered and tested negative for SARS-CoV-2 on PCR.

C2 was an 18–23-year-old male with a past medical history of asthma and a non-specific anxiety disorder who presented to the hospital 28 days prior to study sample collection with signs and symptoms of COVID-19. He was confirmed positive for SARS-CoV-2, admitted, and over the course of ~2 weeks in the hospital recovered with treatment. He was sampled for the study when he was seen in the outpatient setting two weeks later. He was at that time both symptom-free and negative for SARS-CoV-2 infection on PCR.

C3 was a 0–5-year-old male with a past medical history of warm autoimmune hemolytic anemia who presented 2 days prior to study sample collection with signs and symptoms concerning for COVID-19 and was admitted for nine days during which he recovered with treatment. He was sampled for the study both in-hospital during his acute illness, as well as the following discharge at his outpatient clinic visits. For these latter time points, he was no longer symptomatic for COVID-19 but remained infected with SARS-CoV-2 as determined by PCR.

C4 was a 12–17-year-old female with a past medical history of pineoblastoma followed closely in the oncology clinic who reported congestion and smell changes at her outpatient chemotherapy visit and tested positive for SARS-CoV-2 infection that same day. She was sampled for the study roughly weekly starting that day for 5 weeks. During this time her clinical status as it relates to COVID-19 was described by her treating physicians as either mild (i.e., limited to smell changes) or asymptomatic altogether. She remained positive for SARS-CoV-2 infection at all sampling time points until the final week, and also was confirmed positive for SARS-CoV-2 antibody titers at the third sampling time point.

C5 was an 18–23-year-old male with B-cell acute lymphoblastic leukemia followed closely at the oncology clinic who was diagnosed with COVID-19 in the outpatient setting 36 days prior to study sampling. He was managed at home for nearly 3 weeks, at which point, upon coming to the oncology clinic for routine chemotherapy, was found to be febrile, neutropenic, and still positive for SARS-CoV-2. He was admitted to the hospital for COVID-19 treatment, where he recovered over the course of a week. He was first sampled for the study at an outpatient clinic visit 10 days after discharge, by which time he was no longer experiencing COVID-19 symptoms. He was confirmed PCR negative for SARS-CoV-2 during the study period between the first and second sampling time points.

C6 was a 6–11-year-old boy with a past medical history of gastroschisis complicated by short gut syndrome following two small bowel transplants who was hospitalized 25 days prior to study sampling for COVID-19. He recovered with treatment over the course of nine days, was discharged, and then was readmitted about one week later for concerns of rejection of a recent small bowel transplant. He was sampled for the study 8 days into this second admission, at which time he was presumed to be positive for SARS-CoV-2 (based on the test result from day of admission a week earlier) but clinically was no longer exhibiting signs or symptoms of COVID-19.

C7 was a 12–17-year-old male with a past medical history of inflammatory bowel disease who was admitted to the hospital for an inflammatory bowel disease flare. He was tested for SARS-CoV-2 despite not having any signs or symptoms of COVID-19 because at the time it was standard hospital policy to test all admissions for the virus. The patient tested positive for infection but never displayed any symptoms, and also tested positive for SARS-CoV-2 antibodies over the course of his care. The date of study sample collection for this case was not recorded due to a clerical error.

### Control cohort description

Recruitment of age-matched healthy controls (HCs) was not feasible during the study period due to the risk of exposing healthy children to COVID-19 by coming to the hospital for a research blood draw. HCs for this study, therefore, consisted of healthy adults working on-site during the study period (*N* = 4, mean age = 30.75, standard deviation = 6.18).

### RNA extraction

Peripheral blood was collected from study participants into Tempus RNA Blood Tubes (ThermoFisher). As soon as possible after blood collection, tubes were stored at −80 °C until RNA extraction. The MagMax protocol for Stabilized Blood Tubes RNA Isolation Kit (Life Technologies) was used to extract total RNA from 3 mL of collected peripheral blood, following the manufacturer’s instructions. Briefly, frozen blood tubes were thawed to 4 °C prior to RNA extraction. In a biosafety level 2+ (BSL2+) cabinet using proper personal protective equipment (PPE), the blood was first diluted with 1× PBS, then pelleted and washed at 4 °C. The washed and re-pelleted RNA was then dissolved in resuspension solution followed by a dual proteinase and DNase treatment. Next, the RNA was purified through a series of magnetic bead-based washes and eluted in 80 μl of elution buffer as described in the user manual, prior to downstream quantification and library preparation steps. In addition, these modifications to the protocol were followed during extraction: (1) all processing was performed inside a BSL2+ cabinet until resuspension solution was added to the samples, (2) samples were kept on ice throughout the protocol, (3) wash 1 buffer, wash 2 buffer, and isopropanol were kept on ice and used cold, (4) samples were shaken on a vortex adapter at a setting of 4.

### RNA-seq library preparation and sequencing

Total RNA was examined for quantity and quality using the fragment analyzer (Agilent) and Quant-It RNA (ThermoFisher) systems. RNA samples with sufficient material (>50 ng) were passed to whole-transcriptome library preparation using the TruSeq Stranded Total RNA Library Prep Gold (Illumina) following the manufacturer’s instructions. The kit is specifically designed to remove highly abundant ribosomal, globin, and mitochondrial RNA (r- globin- and mt-RNA) from whole blood-derived total RNA in order to enrich for both protein-coding and non-coding RNAs, including microRNAs. Briefly, total RNA inputs were normalized to 100 ng/10 µL (if 100 ng were not available, input amounts were utilized as low as 50 ng) going into preparation. Total RNA was first depleted of highly abundant rRNA, mtRNA, and globinRNA species, prior to enzymatic fragmentation and cDNA generation. The 3**′** ends of cDNA were then adenylated prior to ligation with adapters utilizing unique dual indices (96 UDIs) to barcode samples to allow for efficient pooling and high throughput sequencing. Libraries were enriched with PCR, with all samples undergoing 15 cycles of amplification prior to purification and pooling for sequencing. Completed libraries were quantified using Quant-It reagents and equimolar pools were generated and sequenced on the NovaSeq 6000 using Sprime flow cells with 100 base pair paired-end reads, generating a mean of 50 million paired-end reads per sample.

### RNA-seq data processing, quality control, and normalization

Once sequencing data collection was completed on the instrument, and base calls were converted into raw reads, these raw reads were filtered after quality assessment. The quality-filtered raw data was then converted into FASTQ files using the bcl2fastq Conversion tool (Illumina). RNA-seq reads were aligned to the GRCh38 primary assembly with Gencode release annotation by STAR (v2.7.3a)^48^ using per-sample 2-pass mapping (–twopassMode Basic) and chimeric alignment options (–chimOutType Junctions SeparateSAMold -chimSegmentMin 15 -chimJunctionOverhangMin 15). RNA-seq QC metrics were calculated by fastqc (v0.11.8) and Picard Tools (v2.22.3)^49^. Quantification was done at the gene-level with STAR (-quantMode GeneCounts), at the transcript level with kallisto (v0.46.1), and with antisense, specificity using featureCounts^50^ (Subread R package v1.6.3 and strandness option -s 2) for various counting criteria, including gene-level grouping (-t exon -g gene_id), gene-level grouping / primary alignments only/count all overlapped features (-t exon -g gene_id -primary -O), transcript-level grouping (-t exon -g transcript_id), and exon level/count all overlapped features (-t exon -f -O). A customized version of MultiQC^51^ was used to compile and summarize the per-sample statistics from STAR, Picard Tools and featureCounts (i.e. gene-level counts, mtRNA counts, globinRNA counts, etc) into an interactive HTML report.

We next assessed whether there was mislabeling in our cohort. To do so, we combined two levels of information: correspondence of imputed gender from the gene expression data to the clinical data and identity concordance using genetic information derived from the sequencing data using NGSCheckMate^52^. Using these, we were able to confirm that no samples were mislabeled. After removing lowly expressed genes (keeping genes with counts per million >1 in 10% of the samples), we normalized the raw count data of the 22,302 remaining genes using voom from the limma R package^53^.

RNA-seq data is often impacted by unwanted technical and biological variation that needs to be accounted for prior to any analysis, thus we next explored technical and biological drivers of variance in the normalized count data. Canonical correlation analyses were performed and plotted with the canCorPairs and plotCorrMatrix function from the variancePartition R package^54,55^, and permutation p-values were computed with the p.perm function of the CCP R package^56^. These analyses identified a set of technical and biological covariates that explained a substantial fraction of the expression variation in the dataset and were subsequently accounted for using linear mixed modeling in the DE and co-expression network analyses performed. Specifically, the normalized expression data were adjusted for the following covariates using the fitVarPartModel and the get_predictions function of the variancePartition R package version 1.19.6^54,55^: batch, median insert size, RNA Integrity Number, percent chimeras, PF HQ error rate and median CV coverage, and sex. The median CV coverage is defined by Picard Tools^49^ as “the median coefficient of variation (CV) or stdev/mean for coverage values of the 1000 most highly expressed transcripts” and the PF HQ error rate is defined as “the fraction of bases that mismatch the reference in PF HQ aligned reads”. One sample failed to pass all quality controls and was removed from further analyses. Principal components analyses to explore covariate effect on gene expression variance genome-wide were done using the prcomp R function. The batch effect was calculated at a per gene basis using technical replicates sequenced in all batches. Technical replicate measures were combined in a single gene-expression vector by removing the effect of covariates described above followed up by adding back individual effects in the linear model. One individual was removed due to being an outlier with respect to various quality control metrics.

Age is known to contribute to variation in gene-expression^57^ but in this study recruitment of age-matched HCs was impractical for reasons stated above, leading to a strong correlation between age and disease status (Supplementary Fig. 1b). Leveraging published age gene DE signatures from whole blood^58–60^, we identified 23 modules in a co-expression network built from our data after covariate adjustment significantly enriched for genes associated with age (Supplementary Data 5). To account for this age effect on gene-expression variation without masking the true MIS-C disease signature, we generated a metric for each individual in our cohort to capture the transcriptional effects of age. Using the co-expression network defined above, we extracted all genes in any module significantly enriched for age DE signatures and generated a cross-module eigengene value (Spearman’s rho for the correlation of this metric with age = −0.32, *p*-value = 0.047). We observed that it had an effect on the general patterns of expression in our data without confounding disease state, as illustrated by principal components analyses before and after inclusion of imputed age in the model (Supplementary Fig. 2a, b, respectively*)*. This eigengene was included as an additional covariate in all DE and co-expression network analyses performed below (Supplementary Figs. 1c, d, and 2).

### Cell type deconvolution

Cell type deconvolution was performed with the CIBERSORTx software, using transcripts per million as input and following procedures recommended by the developers^61^. To ensure that we capture signals that are driven by cell-type compositions and not by any single reference used, we used three references generated independently (i.e., by different groups, with different technologies) for this analysis. The LM22 reference^62^ was generated by sorting PMBCs with fluorescent activated cell sorting (FACS) and performing bulk RNA-seq. The NSCLC PBMC^62^ and SCP424^63^ references were generated by single-cell RNA-seq experiments from PBMCs. For SCP424, prior processing had resulted in an unacceptably large range in total UMI present in published cells, with the range spanning over three orders of magnitude (17–53,562 total UMI in individual cells). We, therefore, removed cells with <630 observed genes or less than 1584 total UMI. Furthermore, to account for differences in chemistry or capture efficiency across cells, we performed the simple but lossfull procedure of UMI downsampling to bring all cells to the same level of 1584 total UMI.

We used the lm function in R to test for differences in cell type compositions between disease groups while adjusting for the age cross-module eigengene and for sex. For each cell type in each reference, pairwise comparisons were performed between MIS-C, pediatric COVID-19 and HCs. For each phenotype comparison, *p*-values were adjusted for multiple testing separately for each reference using the false discovery rate (FDR) estimation method of Benjamini–Hochberg^64^ for the number of cell types profiled in the reference.

### Validation of cell-type proportion estimates from deconvolution

To validate the cell type proportion differences observed in deconvolution analyses, we correlated estimated cell type fractions with complete blood counts (CBCs) performed by the clinical laboratory as standard-of-care during hospitalization using Pearson correlation (cor.test R function). For each reference used in cell type deconvolution, p-values were adjusted for multiple testing using the FDR estimation method of Benjamini–Hochberg^64^ for the number of validation tests in the reference. To compare lymphocytes from the CBCs with deconvolution estimates, we derived a single lymphocyte estimate for each of the three cell type references used for deconvolution by summing all T- and B-cell type fractions. Specifically, for NSCLC, we added fraction estimates for T cells CD8, T cells CD4 and B cells; for SCP424 CD4+_T_cell, Cytotoxic_T_cell and B_cell; and for LM22 B cells naive, B cells memory, Plasma cells, T cells CD8, T cells CD4 naive, T cells CD4 memory resting, T cells CD4 memory activated, T cells follicular helper, T cells gamma delta and T cells regulatory (Tregs). For SCP424, monocyte composition was computed by adding fractions of CD16+_monocyte and CD14+_monocyte. Significant correlations were observed between the CBC result and LM22 estimates for neutrophils (Spearman’s rho = 0.83, adjusted *p*-value = 0.011) and monocytes (Spearman’s rho = 0.86, adjusted *p*-value = 0.007). The deconvolution-derived estimates for lymphocytes were significantly correlated with the CBC for all three references (LM22 Spearman’s rho = 0.77, adjusted *p*-value = 0.015; SCP424 Spearman’s rho = 0.78, adjusted *p*-value = 0.028; NSCLC Spearman’s rho = 0.89, adjusted *p*-value = 0.003) (Supplementary Fig. 3d).

### Differential expression

To gain insight into the molecular pathology of MIS-C, we performed DE analyses comparing each phenotype pair in our dataset (Fig. 1c). In order to retain power while accurately controlling the false positive rate it was essential to account for the repeated measures in pediatric COVID-19 cases and the technical replicates in HCs. Towards this end, we used dream^55^, which models the effect of the individual on gene expression as a random effect for each gene using a linear mixed model. We ran three DE analyses in our cohort: MIS-C cases compared to HCs, pediatric COVID-19 cases compared to HCs, and MIS-C compared to pediatric COVID-19 cases (Supplementary Data 15). Covariates included in the model were as described above. Multiple testing was controlled separately for each DE comparison accounting for the 22,302 genes tested using the FDR estimation method of Benjamini–Hochberg^64^.

### Pathway enrichment analyses for DE signatures and co-expression modules

Enrichment for GO, C7, and Hallmark pathways (*N* = 10,192, 4872, and 50 pathways, respectively) was carried out for the upregulated and downregulated signatures from three DE analyses (MIS-C compared to HCs, pediatric COVID-19 compared to HCs, and MIS-C compared to pediatric COVID-19) and the 37 modules from the co-expression network, for a total of 43 feature sets of interest. For each pathway in each database, enrichment was tested as the overlap between the genes in the pathway and the genes in the feature set using as background the 22,302 genes expressed in the dataset. GO analyses were done using the R packages goseq, topGO, and org.Hs.eg.db while C7 and Hallmark analyses were done using the R packages HTSanalyzeR^65^, GSEABase, and GAGE^66^. For each feature set, multiple testing correction for the number of pathways was carried out separately for each pathway database using the FDR estimation method of Benjamini–Hochberg implemented in R using the p.adjust() function.

### Co-expression network analyses

DE analyses permit the broad detection of genes and pathways associated with disease states. Co-expression network analysis builds on this by leveraging the correlation across the transcriptome to organize genes into functional units called modules, sometimes linked to specific cell types and biological processes^67^. All co-expression analyses were performed using weighted gene correlation network analysis (WGCNA) R package version 1.69^13^. Co-expression network modules were created using residual gene-expression of all 30 samples after accounting for biological and technical covariates described above. Standard practices were followed to construct this network, with a soft-power threshold of eight and a minimum module size of 30 genes.

### Co-expression module annotation for cell type and disease expression signatures

To maximize the interpretability of co-expression networks, modules can be annotated to the biological pathways, cell types, and disease processes they capture. Annotation in this context consists of projecting a feature set of interest onto the network and performing an enrichment test for the overlap between the features in the set and the genes in the module. Feature sets tested here included gene sets from pathway databases (see above), cell type markers, and DEGs from our DE analyses and the literature (Figs. 1e,  3, Supplementary Data 10–12, and Supplementary Fig. 4)^12,14^.

To evaluate whether co-expression modules were representative of the activity of specific cell types, we utilized several reference datasets from the literature, each intended to provide a different level of insight into the molecular biology at play within our network, that are described in the Methods. A total of 116 cell type expression profiles from the literature were evaluated for enrichment in the 37 modules and the set of genes that did not fall into a module in our co-expression network separately. Only an upregulated signature was provided for all but 1 cell type, for a total of 117 cell type DE signatures tested. Therefore, study-wide, we performed 4446 tests of enrichment for cell-type signatures in co-expression network modules. Further details on the cell type signatures utilized can be found in the Methods.

To evaluate whether co-expression modules captured the pathology of certain diseases, we utilized several reference datasets from the literature, each intended to provide a different level of insight into the molecular biology at play within our network. A total of 38 disease DE signatures were evaluated: 3 signatures from our data (MIS-C vs. HCs, MIS-C vs. pediatric COVID-19, pediatric COVID-19 vs. HCs) and 35 published disease signatures. The upregulated and downregulated DE signatures were projected onto the 38 modules separately (37 modules and the set of genes that did not fall into a module in our co-expression network), with the exception of 1 dataset that only had an upregulated disease signature for COVID-19. This totaled 75 disease DE signatures tested. Thus, study-wide, a total of *N* = 2850 tests of disease signature enrichment in co-expression modules were performed. Further details on the disease signatures utilized can be found in the Methods.

For every combination of module and DE signature (whether cell type or disease DE signature), we performed a Fisher’s exact test to evaluate enrichment of the module for the DEGs using all 22,302 genes expressed in our dataset as background, with the null hypothesis being that module and DE signature membership are independent. To account for multiple tests, we used a stringent two-step process that was performed separately for cell type and disease DE signature analyses. First, we used a Bonferroni correction to adjust the Fisher’s exact test *p*-value for the number of modules (*N* = 37 plus one group of genes that did not fall into any module) against which each DE signature was tested. We did so because gene-module memberships are unique and therefore all enrichments tests are independent from one another. These adjusted p-values were further corrected for the number of DE signatures tested (*N* = 75 for the disease enrichments and *N* = 117 for the cell type enrichments) using the method of Benjamini–Hochberg to control the FDR. This was done because, unlike for gene-module memberships, DE signatures are highly correlated (e.g., COVID-19 signatures were used from multiple sources, CD8^+^ T cells from multiple sources).

### Age validation

Using gene expression from an age-matched cohort^15^ generated with the same technology (RNA-seq), we replicated the DE and follow-up module enrichment analyses. This cohort was comprised of 40 individuals that had gene expression measures before and after a peanut challenge. For the purpose of this analysis, we only utilized data from unchallenged samples (*N* = 40). To ensure that the identification of the enrichment of skyblue for downregulated genes in MIS-C was independent from age, and unique to MIS-C, we performed DE analyses between these age-matched controls and each group in our study (MIS-C, pediatric COVID-19 and HCs) (Supplementary Data 13 and 14). Following the procedure described above (section titled “RNA-seq data processing, quality control and normalization”) only genes with counts per million >1 in 10% of the samples were included in these analyses, and the raw count data was normalized using voom from the limma R package^53^. We ran DE analyses as described above (section titled “Differential expression”). To minimize unwanted accountable expression variation across the datasets, we included the following covariates in the DE analyses: age and sex. Additionally, we ran DE analyses following the same procedure on 10 randomly downsampled sets of age-matched HCs to match the effective HC sample size of our dataset (*N* = 4, Supplementary Data 13–14). We then projected all the resulting up- and downregulated DEG sets separately onto the co-expression modules following the procedure described above (section titled “Co-expression module annotation for cell type and disease expression signatures”) to identify modules significantly enriched for DEGs identified here.

### CyTOF validation

CyTOF analysis was performed as previously described^68^. Briefly, fresh whole blood samples were stained using the Fluidigm MaxPar Direct Immune Profiling Assay and then fixed and frozen using SmartTube Prot1 buffer. Batched samples were then thawed, red blood cells were lysed and samples were post-fixed and stained with 125 nM Iridium (Ir) intercalator in 2.4% PFA in PBS with 0.08% saponin. Immediately before the acquisition, samples were washed and resuspended in Fluidigm Cell Acquisition Buffer and acquired on a Helios mass cytometer. After the acquisition, data were normalized using the Fluidigm software and analyzed by manual gating using Cytobank. CyTOF was performed on 6/8 MIS-C individuals from our main cohort and on a set of 9 HCs (including 2 HCs from our main cohort) with comparable demographics.

### KD expression signature

To generate a KD DE signature we utilized signatures from Wright et al. JAMA Pediatrics, 2018[16]. The authors performed microarray-based gene expression profiling of whole blood in children with KD and pediatric HCs. Data were downloaded and processed by adapting a script generated from the GEO2R (https://www.ncbi.nlm.nih.gov/geo/geo2r/https://www.ncbi.nlm.nih.gov/geo/geo2r/) web interface. In brief, we downloaded the GSE73464 dataset (47,323 probes and 692 samples) using the getGEO function in the GEOquery package in R^69^. The phenotype table included in the download was queried to identify individuals with a “Discovery” value in the “dataset:ch1” column and either “KD” (*N* = 78) or “Control” (*N* = 55) in the “category:ch1” column. The expression matrix, initially composed of Illumina-calculated signal intensities, was log2 transformed. One KD case (GSM1894671) was excluded for having missing values at every probe. Probes with missingness greater than 90% in either the KD cases or the controls were removed, as were probes where the ratio of missingness between cases and controls was greater or less than one standard deviation away from the mean missingness ratio^69^.

We were unable to reproduce the DE analysis exactly as performed in the original publication because the public data release did not contain the age covariate used in the model in the paper. Performing DE using the lmFit and Bayes functions in the limma R package^53^ without accounting for any covariates resulted in 3310 DEGs by the criteria of the original publication (absolute value of the log_2_fold-change >1). This finding was inconsistent with the DE results reported in the original publication. In that report, DE was performed between KD cases and 5 other comparator groups (HCs plus pediatric cohorts with 4 types of febrile illnesses). Transcriptome-wide DE results comparing KD to HCs were not described, but it was noted that 1600 transcripts were identified as differentially expressed between KD and ≥1 of the 5 comparator groups (the number of DEGs between KD and each comparator group was not to our knowledge reported). Though only performing DE between 1 of the 5 comparator groups we observed over 2 times the number of DEGs, leading us to conclude that our analysis was identifying a large number of false positives due to our inability to correct for confounding variables such as age. We, therefore, sought to impose a more conservative definition of DE between KD and controls. We evaluated the number of differentially expressed transcripts surviving different thresholds of the −log_10_(adjusted *p*-value). Our rationale in doing so was to identify the threshold that returned a number of DE transcripts that was comparable to what was found in the original report. Since the number of DE transcripts between KD and HCs was not reported in the original publication, we assumed that ~50% of the 1600 DE transcripts identified were observed in the analysis comparing KD to HCs, and therefore selected the −log_10_(adjusted *p*-value) threshold that identified ~800 DE transcripts (−log10(adjusted *p*-value) >9; 831 DE transcripts). After selecting this threshold, Illumina probe identifiers were mapped to Ensembl gene identifiers using the illuminaHumanv4.db R package^70^. For any Ensembl gene represented by more than one probe we retained the probe with the most significant unadjusted *p*-value, resulting in 662 DEGs (599 upregulated and 63 downregulated in KD).

### MAS and NOMID expression signatures

MAS and NOMID signatures were obtained from Canna et al. *Nature Genetics*, 2014^17^: The authors performed whole blood bulk RNA-seq to define DE signatures for MAS (*N* = 1, 7 time points) and NOMID (*N* = 2, sampled before and after treatment with anakinra) compared to healthy controls (HCs; *N* = 5). DE signatures for MAS (compared to HCs) and NOMID pre-treatment (compared to HCs) were defined in the original publication as the top 50 upregulated and downregulated genes. For some of these signatures, *p*-values were reported, while for others, they were not. For our analyses we elected to treat all 50 genes as DEGs for each signature. The original publication reported results at the transcript level, and after mapping to Ensembl gene identifiers using HUGO^71^ we noted that for some genes, different transcripts were reported as differentially expressed in opposite directions for the same condition. These genes were excluded from our analyses, leaving 89 MAS DEGs (42 upregulated and 47 downregulated) and 65 NOMID DEGs (33 upregulated and 32 downregulated) in our analyses.

### Autoimmune expression signatures

To define the expression signature of autoimmunity we utilized data from Barturen et al. *medrxiv*, 2020^23^: These authors performed bulk RNA-seq of whole blood in seven systemic autoimmune diseases from 918 patients and 263 HCs. The seven systemic autoimmune diseases were undifferentiated connective tissue disease (UCTD), systemic lupus erythematosus (SLE), rheumatoid arthritis (RA), mixed connective tissue disease (MCTD), systemic sclerosis (SSC), Sjögren’s syndrome (SJS), and primary antiphospholipid syndrome (PAPS). DEGs were defined as adjusted *p*-value < 0.05 in the summary statistics provided by the authors upon request. The following number of DEGs were included for each condition: 79 for UCTD (56 upregulated and 23 downregulated), 480 for SLE (287 upregulated and 193 downregulated), 37 for RA (26 upregulated and 11 downregulated), 922 for MCTD (561 upregulated and 361 downregulated), 72 for SSC (43 upregulated and 29 downregulated), 283 for SJS (225 upregulated and 68 downregulated), and 20 for PAPS (8 upregulated and 12 downregulated).

### Annotating skyblue for cytotoxic cell subtypes

To delineate whether the cell types annotated to skyblue were limited to one MIS-C signature direction, we defined “MIS-C upregulated” and “MIS-C downregulated” subsets of skyblue by keeping the genes in the module upregulated or downregulated in MIS-C, respectively. We then projected the CD8^+^ T cell and NK cell signatures that we had found enriched in skyblue earlier onto the upregulated and downregulated subsets. As these were targeted secondary analyses, we did not perform multiple test corrections.

In addition to the primary cell type enrichment analyses, we performed hypothesis-driven follow-up analyses to further delineate the subtypes of CD8^+^ and NK cells enriched in the skyblue module. For these analyses, since they were targeted hypotheses for a single module, we only adjusted *p*-values for the number of DE signatures tested (*N* = 43) using the method of Benjamini and Hochberg. The cell subtype signatures utilized to dissect skyblue were from four literature sources:

Beltra et al. *Immunity*, 2020^27^: The authors performed RNA sequencing of bulk, Ly108- and CD69-sorted blood cells from LCMV-infected mice to define transcriptional signatures of exhausted CD8^+^ T cells (Tex), then linked these subsets in a hierarchical developmental scheme. Four subtypes of Tex were defined, which for clarity they refer to as progenitor 1 (Ly108^+^CD69^+^; Tex^prog1^), progenitor 2 (Ly108^+^CD69^−^; Tex^prog2^), intermediate (Ly108^−^CD69^−^; Tex^int^), and terminal (Ly108^−^CD69^+^; Tex^term^). Functionally, they found the two progenitor Tex states differed, with Tex^prog1^ quiescent and blood inaccessible and Tex^prog2^ capable of initiating cell cycling and gaining access to circulation. Tex^prog2^ gave rise to Tex^int^, which had some effector-like features and ultimately terminally differentiated into Tex^term^ (i.e., permanently exited the cell cycle). For each of the four defined subsets, the authors provided three DE signatures (one for each of the other three subsets) as a matrix where values were either −1, 0, or 1 to indicate downregulation, no DE, and upregulation, respectively. Mouse gene names were mapped to human genes using the biomaRt package in R^73,73^ and all DEGs for each Tex subset were utilized in our enrichment analyses.

Collins et al. *Cell*, 2019^74^: The authors sought to identify transcriptional signatures amongst subtypes of human natural killer (NK) cells in circulation. DEG lists were provided in the supplementary information for pairwise comparisons of CD56^bright^ NK cells and two types of CD56^dim^ NK cells (CD56^dim^CD57^+^ and CD56^dim^CD57^−^). We mapped gene symbols to Ensembl using HUGO^71^ and included all DEGs in our enrichment analyses of the skyblue module.

Wherry et al. *Immunity*, 2007^25^: The authors profiled gene expression using microarrays to determine the molecular signature of exhaustion in CD8^+^ T cells (Tex) compared to memory and effector CD8^+^ T cell subtypes in LCMV-infected mice. Tables of Tex upregulated and downregulated DEGs (compared to both effector and memory CD8^+^ T cells) were provided in the supplementary information in image form and converted into a table using Adobe Acrobat image conversion software. Mouse gene identifiers were then converted to human Ensembl gene identifiers using the biomaRt package in R^72,73^.

Yang et al. *Nature Communications*, 2019^75^: The authors performed scRNA-seq on 3046 NK cells from the whole blood of two healthy donors. NK cells were extracted using CD7, clustered according to transcriptional signatures, and DEGs of each cluster were defined relative to all other clusters (adjusted *p*-value < 0.05). The authors then manually annotated the clusters to define five circulating NK subsets: CD56^bright^, a transitional stage between CD56^bright^ and CD56^dim^ NK cells, an active group of NK cells consisting of mixed developmental stages, and two clusters (“mature and terminal”) that represent functionally mature CD56^dim^CD57^+^ NK cells with the highest cytolytic potential and transcriptional profiles that differ from one another enough to cluster separately in transcriptional space. We used the DEGs for all of these clusters to annotate the skyblue module.

### Cell type and disease expression signatures curated from the literature

Several reference datasets from the literature capturing the transcriptional signature of diseases and cell types were utilized in this report to provide a different level of insight into the molecular biology at play within our co-expression network. A total of 35 disease signatures and 116 cell type signatures from the literature were evaluated. Below we provide information on how each signature was generated in the original publication and utilized in our analyses, with the exception of select signatures that we described in the “Methods” section.

Blanco-Melo et al. *Cell*, 2020^19^: The authors performed a variety of RNA-seq experiments, but we only utilized data from three in our analyses: a comparison of lung samples from two COVID-19 cases and two unaffected controls, a comparison of normal human bronchial epithelial (NHBE) cell lines with and without SARS-CoV-2 infection, and a comparison of NHBE cell lines with and without IAV infection. The full DE signatures were provided in the published manuscript, including both adjusted and unadjusted *p*-values. We defined DEGs by an adjusted *p*-value below 0.05 resulting in 1767 DEGs for the COVID-19 lung tissue signature (527 upregulated and 1240 downregulated in COVID-19), 526 DEGs for the SARS-CoV-2 NHBE signature (367 upregulated and 159 downregulated in SARS-CoV-2 infection), and 2684 DEGs in the NHBE IAV signature (1426 upregulated and 1258 downregulated in IAV infection).

Bojkova et al. *Nature*, 2020^76^: The authors infected Caco-2 cells with either SARS-CoV-2 or a mock infection and quantified translation for 2715 proteins and total protein levels for 6382 proteins by mePROD proteomics at several time points from 2 to 24 h following infection. These signatures were provided in the supplementary information of the publication, and for our analyses, only the total protein signature at 24 h after infection was utilized. UniProt identifiers were mapped to Ensembl using HUGO^71^. In some instances, more than one result was provided for the same protein Ensembl identifier, in which case we kept the result with the strongest association to SARS-CoV-2. DE proteins were defined using a threshold of 0.05 for the p-values provided in the published materials, resulting in 1509 DE proteins (803 upregulated and 706 downregulated).

Gordon et al. *Nature*, 2020^77^: The authors expressed 26 viral SARS-CoV-2 proteins in human embryonic kidney 293 cell lines then used affinity-purification mass spectrometry to identify 332 pathogen–host protein–protein interactions. UniProt identifiers provided in the supplementary information with the publication were mapped to Ensembl identifiers using HUGO^71^ and all human proteins in the 332 interactions were treated as a set of “upregulated” COVID-19 DEGs in our analyses.

Liao et al. *Nature Medicine*, 2020^78^: The authors performed single-cell RNA-seq (scRNA-seq) on cells extracted from bronchoalveolar lavage fluid (BALF) obtained from six severe COVID-19 cases, three moderate COVID-19 cases, three HCs, and one publicly available BALF sample. The only disease transcriptional signature provided in the materials with the publication were DEGs of CD8^+^ T cells in moderate compared to severe COVID-19 patients. After mapping gene symbols to Ensembl identifiers using HUGO^71^, we used for our analyses 224 genes upregulated and 233 genes downregulated in severe compared to moderate COVID-19 CD8^+^ T cells. In addition, we utilized cell type expression signatures from this report to annotate modules in our co-expression network. The authors provided signatures for macrophage and T cell clusters as lists of genes upregulated in the cluster. In addition, they provided lists of genes differentially expressed between expanded and non-expanded CD8^+^ T cells from the BALF of moderate COVID-19 cases. These were provided as two separate lists, which we converted into a single DE signature by defining upregulated genes as those with increased expression in expanded and downregulated genes as those with increased expression in the non-expanded populations.

Liu et al. *Genome Biology*, 2017^79^: The authors performed an RNA-seq of whole blood from fatal cases of EVD (*N* = 88) compared to a group of recovered EVD cases (*N* = 16). DE results were provided in the supplementary information of the publication and we defined DEGs using the criteria set forth by the authors in the text of their manuscript (an adjusted *p*-value < 0.05 and absolute value of the log_2_(fold-change) > 1). This led to 1492 DEGs (1350 upregulated and 142 downregulated).

Newman et al. *Nature Methods*, 2015^80^: The authors designed a leukocyte gene expression signature matrix, termed “LM22”, that contains 547 genes distinguishing 22 human hematopoietic cell types from one another at the transcriptional level. These include several T cell subtypes, naïve and memory B-cells, plasma cells, NK cells, and multiple myeloid subsets. These signatures were created by the authors by collating gene expression profiles from the public domain. Each gene was treated as upregulated for the given cell type in our enrichment analyses.

Park et al. *Science*, 2020^81^: The authors performed scRNA-seq on dissociated cells from the human thymus sampled from 15 embryonic and fetal thymi (spanning thymic developmental stages between seven and 17 post-conception weeks) and nine postnatal thymi from pediatric and adult individuals. They annotated cells into 55 types or states, and for each cell type or state provided a list of the top 20 marker genes. We utilized these markers as DEGs for cell types and states in our analyses after mapping gene symbols to Ensembl gene identifiers using HUGO^71^, treating all genes as upregulated in the given cell type or state.

Ramilo et al. *Blood*, 2007^82^: The authors performed microarray gene expression profiling of blood leukocytes from 11 individuals with acute IAV infection and 12 individuals with an acute bacterial infection. DE results for the genes the authors had defined as DEGs were provided in the supplementary information of the publication as log_2_(fold-change) values and unadjusted *p*-values. For genes with multiple probes, we retained the probe with the most significant association to IAV infection. After mapping to Ensembl gene identifiers using HUGO^71^, 459 DEGs were included in our analyses (211 upregulated and 248 downregulated in IAV infection).

Rowley et al. *Blood*, 2011^83^: The authors performed bulk RNA-seq of platelets isolated from the blood of two human donors. A table is provided in the manuscript of the most highly expressed non-ubiquitous genes in these platelets. After mapping gene symbols to Ensembl identifiers using HUGO^71^, we treated these genes as a platelet DE signature in our enrichment analyses.

Shen et al. *Cell*. 2020^84^: The authors performed proteomic and metabolomic profiling of sera from severe COVID-19 cases (*N* = 28), non-severe COVID-19 cases (*N* = 25), “non-COVID-19” cases who had a clinical picture suggestive of COVID-19 but tested negative for SARS-CoV-2 (*N* = 25), and HCs (*N* = 28). For our analyses, we used the four proteomic DE signatures provided with the supplementary information of the publication: severe COVID-19 vs. HCs, non-severe COVID-19 vs. HCs, severe COVID-19 vs. non-severe COVID-19, and non-COVID-19 vs HCs. UniProt protein identifiers were mapped to Ensembl gene identifiers using HUGO^71^. Summary statistics provided were limited to genes the authors found differentially expressed and included both adjusted and unadjusted p-values. We defined DEGs using an adjusted *p*-value threshold of 0.05, resulting in 29 DEGs for non-COVID-19 compared to HCs (16 upregulated and 13 downregulated), 41 DEGs for severe COVID-19 compared to non-severe COVID-19 (25 upregulated and 16 downregulated), 43 DEGs for non-severe COVID-19 compared to HCs (20 upregulated and 23 downregulated), and 116 DEGs for severe COVID-19 compared to HCs (58 upregulated and 58 downregulated).

Szabo et al. *Nature Communications*, 2019^85^: The authors performed scRNA-seq on >50,000 resting and activated human T cells isolated from lungs, lymph nodes, bone marrow, and blood from two deceased adult donors who had no history of any chronic disease or cancer. T cell DE profiles were derived by the authors using a novel method that identifies expression patterns called factors that represent discrete or continuous biological processes and downloaded with the supplementary information from the publication. Mapping of gene symbols to Ensembl gene identifiers was performed using HUGO^71^. All markers were treated as upregulated in the given cell type or state in our enrichment analyses.

Thair et al. medRxiv, 2020^86^: The authors performed RNA-seq on whole blood from 62 COVID-19 cases and 24 healthy controls. A total of 1998 DEGs were provided in the supplementary information of the preprint (768 upregulated and 1230 downregulated).

Wen et al. *Cell Discovery*, 2020^87^: The authors performed scRNA-seq on 70,858 PBMCs from 10 COVID-19 patients, five of which were defined as being in the early recovery stage (ERS) and five in the late recovery stage (LRS), compared the resulting expression profiles to scRNA-seq of 57,238 PBMCs from five HCs. No DE results were provided in table form in this publication, so we assembled DEG lists manually by copying into a table genes labeled as DEGs in the volcano plots in the supplementary information of the publication. The authors provided three such plots in their supplementary information: antibody-secreting cells (ASC) in ERS compared to HC, dendritic cells (DCs) in ERS compared to HCs, and proliferating T cells in ERS compared to HCs. After gene symbols from the plots were mapped to Ensembl identifiers using HUGO^71^, we included in our analyses 33 DEGs found in ASCs in ERS compared to HCs (25 upregulated and eight downregulated), 31 DEGs found in proliferating T cells in ERS compared to HCs (29 upregulated and two downregulated), and 69 DEGs found in DCs in ERS compared to HCs (665 upregulated and four downregulated).

Wilk et al. *Nature Medicine*, 2020^88^: The authors performed scRNA-seq on PBMCs from seven patients hospitalized with COVID-19 and six HCs. In the supplementary information, DE results for COVID-19 within seven different cell types were provided: CD14^+^ monocytes, CD16^+^ monocytes, DCs, NK cells, CD8^+^ T cells, CD4^+^ T cells, and B-cells. Results are provided only for genes defined by the authors as COVID-19 DEGs for the given cell type. DEGs were defined by the authors on a per-individual basis, requiring an absolute value of log_2_(fold-change) >0.25 and an adjusted *p*-value < 0.05 from an analysis comparing the cells from that case to the cells from all HCs. No *p*-values or other summary statistics were included in the published material. For genes that were DEGs in one case but not another, the log_2_(fold-change) value for the former individual was set to 0 in the tables provided. For many genes, it was reported as upregulated in one individual and downregulated in another for the same cell type. Given the format of the results provided, we sought to reduce the data to a single summary statistic for each gene in each cell type. To do this, we averaged the log_2_(fold-change) values across individuals, including the zeroes when genes were not DEGs, and treated any gene with a mean value greater or less than zero as upregulated or downregulated, respectively. After implementing these steps, the following number of DEGs between COVID-19 and HCs were found: 383 DEGs in CD14^+^ monocytes (208 upregulated and 175 downregulated), 248 DEGs in CD16^+^ monocytes (204 upregulated and 44 downregulated), 222 DEGs in DCs (180 upregulated and 42 downregulated), 164 DEGs in NK cells (95 upregulated and 69 downregulated), 171 DEGs in CD8^+^ T cells (99 upregulated and 72 downregulated), 164 DEGs in CD4^+^ T cells (103 upregulated and 61 downregulated), 450 DEGs in B-cells (253 upregulated and 197 downregulated). In addition to using the COVID-19 DE signatures from this publication, we also used the cell type signatures provided in the supplementary materials of this publication to annotate our co-expression network modules. For 20 different cell types, the authors provided a list of genes upregulated in that cell type compared to the 19 other cell types in the dataset. Genes with an adjusted *p*-value below 0.05 for a given cell type were defined as markers for that cell type in our analyses.

Xiong et al. *Emerging Microbes and Pathogens*, 2020^89^: The authors performed bulk RNA sequencing comparing gene expression in BALF from COVID-19 cases (*N* = 2) and HCs from a previous study (*N* = 3) as well as PBMCs from COVID-19 cases (*N* = 3) and HCs (*N* = 3). Both the BALF and PBMC COVID-19 signatures were obtained by downloading the supplementary information provided with the published manuscript, which included summary statistics for each gene. To define DEGs, we used an adjusted *p*-value threshold of 0.05, resulting in 5304 COVID-19 DEGs in BALF (2952 upregulated and 2,352 downregulated) and 2082 COVID-19 DEGs in PBMCs (1421 upregulated and 661 downregulated).

### MIS-C modules projected onto Bayesian networks

Whereas co-expression networks primarily capture linear relationships between genes, they do not account for more complex, nonlinear, and statistically inferred causal regulatory relationships. Bayesian networks (BNs) are graphical models useful for the latter and have proven a valuable framework for capturing the flow of information within high-dimensional molecular data. The rarity of MIS-C did not allow sufficient samples to construct a causal network for this disease directly. Instead, we identified a recently constructed BN from a large IBD cohort comprised of 209 healthy controls, 389 ulcerative colitis patients, and 432 Crohn’s disease patients from whom blood was collected and RNA sequenced^30^ that will be reported in an independent publication (in review). This network was constructed using RIMBANET^90–92^.

We examined the 11 MIS-C modules and cyan by projecting them onto the BN, identifying all genes within one path length of these module genes and the largest connected subnetwork from these projections as the representation of the module in the BN. We performed KDA on each of the MIS-C module subnetworks to identify those genes predicted by the BN to regulate the module (Supplementary Data 18). KDA was performed using the Key Driver Analysis R package^31^ in which a key driver is defined as a gene that is significantly upstream and connected to a set of genes of interest (i.e., a module).

### Reporting summary

Further information on research design is available in the Nature Research Reporting Summary linked to this article.

## Supplementary information

Supplementary Information

Reporting Summary

Description of Additional Supplementary Files

Supplementary Data 1-21

## Data Availability

The datasets generated during and/or analyzed during the current study have been deposited in GEO: https://www.ncbi.nlm.nih.gov/geo/query/acc.cgi?acc=GSE178388. The datasets generated during and/or analyzed during the current study are also available on GEO. Due to risk of re-identification, minimal phenotype data can be provided outside of an indicator of case/control status. Specimens collected for this study may be requested by contacting the authors and could be made available depending on the amount of remaining samples. Source data are provided with this paper.

## Source data

Source Data

## Code availability

No custom code on unpublished methods was developed for the study presented here. All code used in the generation of this manuscript is available upon requests.

## References

[1] Dufort EM (2020). Multisystem inflammatory syndrome in children in New York State. N. Engl. J. Med..

[2] Riphagen S, Gomez X, Gonzalez-Martinez C, Wilkinson N, Theocharis P (2020). Hyperinflammatory shock in children during COVID-19 pandemic. Lancet.

[3] Center for Disease Control and Prevention.* HAN Archive-00432|Health**Alert Network (HAN)*. https://emergency.cdc.gov/han/2020/han00432.asp (2020).

[4] Verdoni L (2020). An outbreak of severe Kawasaki-like disease at the Italian epicentre of the SARS-CoV-2 epidemic: an observational cohort study. Lancet.

[5] Center for Disease Control and Prevention. *Kawasaki Syndrome* https://www.cdc.gov/kawasaki/index.html (2020).

[6] Uzhachenko RV, Shanker A (2019). CD8 T lymphocyte and NK cell network: circuitry in the cytotoxic domain of immunity. Front. Immunol..

[7] Cook KD, Whitmire JK (2013). The depletion of NK cells prevents T cell exhaustion to efficiently control disseminating virus infection. J. Immunol..

[8] Waggoner SN, Cornberg M, Selin LK, Welsh RM (2011). Natural killer cells act as rheostats modulating antiviral T cells. Nature.

[9] McKinney EF, Lee JC, Jayne DRW, Lyons PA, Smith KGC (2015). T cell exhaustion, co-stimulation and clinical outcome in autoimmunity and infection. Nature.

[10] Wu D, Smyth GK (2012). Camera: a competitive gene set test accounting for inter-gene correlation. Nucleic Acids Res..

[11] Storey JD, Tibshirani R (2003). Statistical significance for genomewide studies. Proc. Natl Acad. Sci. USA.

[12] Subramanian A (2005). Gene set enrichment analysis: a knowledge-based approach for interpreting genome-wide expression profiles. Proc. Natl Acad. Sci. USA.

[13] Langfelder, P. & Horvath, S. WGCNA: an R package for weighted correlation network analysis. *BMC Bioinform.* **9**, 559, (2008). 10.1186/1471-2105-9-559 PMC2631488 19114008

[14] Ashburner M (2000). Gene ontology: tool for the unification of biology. The Gene Ontology Consortium. Nat. Genet..

[15] Watson CT (2017). Integrative transcriptomic analysis reveals key drivers of acute peanut allergic reactions. Nat. Commun..

[16] Wright VJ (2018). Diagnosis of Kawasaki disease using a minimal whole-blood gene expression signature. JAMA Pediatr..

[17] Canna SW (2014). An activating NLRC4 inflammasome mutation causes autoinflammation with recurrent macrophage activation syndrome. Nat. Genet..

[18] Acharya D, Liu G, Gack MU (2020). Dysregulation of type I interferon responses in COVID-19. Nat. Rev. Immunol..

[19] Blanco-Melo, D. et al. Imbalanced host response to SARS-CoV-2 drives development of COVID-19. *Cell* **181**, 1036–1045 e1039 (2020). 10.1016/j.cell.2020.04.026 PMC7227586 32416070

[20] Gruber, C. et al. Mapping Systemic Inflammation and Antibody Responses in Multisystem Inflammatory Syndrome in Children (MIS-C). *Cell* **183**, 982–995 e914, (2020). 10.1016/j.cell.2020.09.034 PMC7489877 32991843

[21] Cavounidis, A., Alderson, J. & Quastel, M. Multisystem inflammatory syndrome in children: getting to the heart of the matter. *Nat. Rev. Immunol*. 10.1038/s41577-020-0409-z (2020). 10.1038/s41577-020-0409-z PMC7391040 32732952

[22] Consiglio, C. R. et al. The Immunology of Multisystem Inflammatory Syndrome in Children with COVID-19. *Cell* **183**, 968-981 e967, (2020) 10.1016/j.cell.2020.09.016 PMC7474869 32966765

[23] Barturen, G. et al. Integrative Analysis Reveals a Molecular Stratification of Systemic Autoimmune Diseases. *Arthritis Rheumatol* **73**, 1073–1085, (2021) 10.1002/art.41610 33497037

[24] Kalia, V. & Sarkar, S. Regulation of effector and memory CD8 T cell differentiation by IL-2—a balancing act. *Front. Immunol.* **9**, 2987, (2018). 10.3389/fimmu.2018.02987 PMC6306427 30619342

[25] Wherry EJ (2007). Molecular signature of CD8+ T cell exhaustion during chronic viral infection. Immunity.

[26] Blank CU (2019). Defining ‘T cell exhaustion’. Nat. Rev. Immunol..

[27] Beltra J-C (2020). Developmental relationships of four exhausted CD8 T cell subsets reveals underlying transcriptional and epigenetic landscape control mechanisms. Immunity.

[28] Michel T (2016). Human CD56bright NK cells: an update. J. Immunol..

[29] Lopez-Vergès S (2010). CD57 defines a functionally distinct population of mature NK cells in the human CD56dimCD16+ NK-cell subset. Blood.

[30] Suárez-Fariñas, M. et al. Intestinal Inflammation Modulates the Expression of ACE2 and TMPRSS2 and Potentially Overlaps With the Pathogenesis of SARS-CoV-2-related Disease. *Gastroenterology* **160**, 287–301 e220, (2021) 10.1053/j.gastro.2020.09.029 PMC7516468 32980345

[31] Zhang, B. & Zhu, J. Identification of key causal regulators in gene networks. In *Proc. World Congress on Engineering*, Vol. 2, 5–8. (eds. S. I. Ao and Len Gelman and David WL Hukins and Andrew Hunter and A. M. Korsunsky), (2013).

[32] Foltz, J. A., Moseman, J. E., Thakkar, A., Chakravarti, N. & Lee, D. A. TGFβ imprinting during activation promotes natural killer cell cytokine hypersecretion. *Cancers* **10**, 423, (2018). 10.3390/cancers10110423 PMC6267005 30400618

[33] Tinoco R, Alcalde V, Yang Y, Sauer K, Zuniga EI (2009). TGF-β signaling in T cells is essential for CD8 T cell suppression and viral persistence in vivo. Immunity.

[34] Wang, P. et al. The transcription factor T-Bet is required for optimal type I follicular helper T cell maintenance during acute viral infection. *Front. Immunol*. **10**, 606, (2019). 10.3389/fimmu.2019.00606 PMC6449430 30984183

[35] He B (2016). CD8 T cells utilize highly dynamic enhancer repertoires and regulatory circuitry in response to infections. Immunity.

[36] Leavy O (2012). Maturation and function of NK cells. Nat. Rev. Immunol..

[37] Drouillard A (2018). S1PR5 is essential for human natural killer cell migration toward sphingosine-1 phosphate. J. Allergy Clin. Immunol..

[38] Jenne CN (2009). T-bet–dependent S1P5 expression in NK cells promotes egress from lymph nodes and bone marrow. J. Exp. Med..

[39] Naluyima, P. et al. Terminal effector CD8 T cells defined by an IKZF2 + IL-7R—transcriptional signature express FcγRIIIA, expand in HIV infection, and mediate potent HIV-specific antibody-dependent cellular cytotoxicity. *J. Immunol*. **203**, 2210–2221, (2019). 10.4049/jimmunol.1900422 PMC6778306 31519862

[40] Roncagalli R (2005). Negative regulation of natural killer cell function by EAT-2, a SAP-related adaptor. Nat. Immunol..

[41] Yeo L (2018). Autoreactive T effector memory differentiation mirrors β cell function in type 1 diabetes. J. Clin. Investig..

[42] Kim JY (2014). A novelPRF1gene mutation in a fatal neonate case with type 2 familial hemophagocytic lymphohistiocytosis. Korean J. Pediatr..

[43] Lee SM, Molleran Lee S (2004). Characterisation of diverse PRF1 mutations leading to decreased natural killer cell activity in North American families with haemophagocytic lymphohistiocytosis. J. Med. Genet..

[44] Zhong H (2017). Whole transcriptome profiling reveals major cell types in the cellular immune response against acute and chronic active Epstein-Barr virus infection. Sci. Rep..

[45] Bongen, E., Vallania, F., Utz, P. J. & Khatri, P. KLRD1-expressing natural killer cells predict influenza susceptibility. *Genome Med*. **10**, 45, (2018). 10.1186/s13073-018-0554-1 PMC6001128 29898768

[46] Fang M (2011). CD94 is essential for NK cell-mediated resistance to a lethal viral disease. Immunity.

[47] Cannons, J. L., Tangye, S. G. & Schwartzberg, P. L. SLAM family receptors and SAP adaptors in immunity. *Annu. Rev. Immunol*. **29**, 665–705, (2011). 10.1146/annurev-immunol-030409-101302 21219180

[48] Dobin A (2013). STAR: ultrafast universal RNA-seq aligner. Bioinformatics.

[49] Broad Institute. Picard Toolkit. *Broad Institute, GitHub repository*, http://broadinstitute.github.io/picard/ (2019).

[50] Liao Y, Smyth GK, Shi W (2014). featureCounts: an efficient general purpose program for assigning sequence reads to genomic features. Bioinformatics.

[51] Ewels P, Magnusson M, Lundin S, Käller M (2016). MultiQC: summarize analysis results for multiple tools and samples in a single report. Bioinformatics.

[52] Lee S (2017). NGSCheckMate: software for validating sample identity in next-generation sequencing studies within and across data types. Nucleic Acids Res..

[53] Ritchie ME (2015). limma powers differential expression analyses for RNA-sequencing and microarray studies. Nucleic Acids Res..

[54] Hoffman GE, Schadt EE (2016). variancePartition: interpreting drivers of variation in complex gene expression studies. BMC Bioinform..

[55] Hoffman GE, Roussos P (2020). dream: powerful differential expression analysis for repeated measures designs. Bioinformatics.

[56] Menzel, U. *CCP: Significance Tests for Canonical Correlation Analysis. Comprehensive R Archive Network.* https://CRAN.R-project.org/package=CCP (2012).

[57] Solana R (2012). Innate immunosenescence: effect of aging on cells and receptors of the innate immune system in humans. Semin. Immunol..

[58] Yang J (2015). Synchronized age-related gene expression changes across multiple tissues in human and the link to complex diseases. Sci. Rep..

[59] Peters MJ (2015). The transcriptional landscape of age in human peripheral blood. Nat. Commun..

[60] Lin H (2019). Whole blood gene expression associated with clinical biological age. J. Gerontol. A.

[61] Steen, C. B., Liu, C. L., Alizadeh, A. A. & Newman, A. M. Profiling cell type abundance and expression in bulk tissues with CIBERSORTx. *Methods Mol. Biol*. **2117**, 135–157, (2020). 10.1007/978-1-0716-0301-7_7 PMC7695353 31960376

[62] Newman AM (2019). Determining cell type abundance and expression from bulk tissues with digital cytometry. Nat. Biotechnol..

[63] Ding, J. et al. Systematic comparison of single-cell and single-nucleus RNA-sequencing methods. *Nat Biotechnol*, **38**, 737–746, (2020). 10.1038/s41587-020-0465-8 PMC7289686 32341560

[64] Hochberg Y, Benjamini Y (1990). More powerful procedures for multiple significance testing. Stat. Med..

[65] Wang X, Terfve C, Rose JC, Markowetz F (2011). HTSanalyzeR: an R/Bioconductor package for integrated network analysis of high-throughput screens. Bioinformatics.

[66] Luo W, Friedman MS, Shedden K, Hankenson KD, Woolf PJ (2009). GAGE: generally applicable gene set enrichment for pathway analysis. BMC Bioinforma..

[67] Langfelder P, Mischel PS, Horvath S (2013). When is hub gene selection better than standard meta-analysis?. PLoS ONE.

[68] Geanon, D. et al. A streamlined whole blood CyTOF workflow defines a circulating immune cell signature of COVID-19. *Cytometry A*, 10.1002/cyto.a.24317 (2021). 10.1002/cyto.a.24317 PMC8013522 33496367

[69] Davis S, Meltzer PS (2007). GEOquery: a bridge between the Gene Expression Omnibus (GEO) and BioConductor. Bioinformatics.

[70] Dunning, M., Lynch, A. & Eldridge, M. *Illumina HumanHT12v4 annotation data*. Bioconductor, (2015).

[71] Eyre TA (2006). The HUGO Gene Nomenclature Database, 2006 updates. Nucleic Acids Res..

[72] Durinck S, Spellman PT, Birney E, Huber W (2009). Mapping identifiers for the integration of genomic datasets with the R/Bioconductor package biomaRt. Nat. Protoc..

[73] Durinck S (2005). BioMart and Bioconductor: a powerful link between biological databases and microarray data analysis. Bioinformatics.

[74] Collins PL (2019). Gene regulatory programs conferring phenotypic identities to human NK. Cells Cell.

[75] Yang C (2019). Heterogeneity of human bone marrow and blood natural killer cells defined by single-cell transcriptome. Nat. Commun..

[76] Bojkova D (2020). Proteomics of SARS-CoV-2-infected host cells reveals therapy targets. Nature.

[77] Gordon DE (2020). A SARS-CoV-2 protein interaction map reveals targets for drug repurposing. Nature.

[78] Liao M (2020). Single-cell landscape of bronchoalveolar immune cells in patients with COVID-19. Nat. Med..

[79] Liu X (2017). Transcriptomic signatures differentiate survival from fatal outcomes in humans infected with Ebola virus. Genome Biol..

[80] Newman AM (2015). Robust enumeration of cell subsets from tissue expression profiles. Nat. Methods.

[81] Park, J. -E. et al. A cell atlas of human thymic development defines T cell repertoire formation. *Science* **367**, eaay3224, (2020). 10.1126/science.aay3224 PMC7611066 32079746

[82] Ramilo O (2007). Gene expression patterns in blood leukocytes discriminate patients with acute infections. Blood.

[83] Rowley JW (2011). Genome-wide RNA-seq analysis of human and mouse platelet transcriptomes. Blood.

[84] Shen B (2020). Proteomic and metabolomic characterization of COVID-19 patient sera. Cell.

[85] Szabo, P. A. et al. Single-cell transcriptomics of human T cells reveals tissue and activation signatures in health and disease. *Nat. Commun.* **10**, 4706, (2019). 10.1038/s41467-019-12464-3 PMC6797728 31624246

[86] Thair, S. A. et al. Transcriptomic similarities and differences in host response between SARS-CoV-2 and other viral infections. *iScience* **24**, 101947, (2021). 10.1016/j.isci.2020.101947 PMC7786129 33437935

[87] Wen W (2020). Immune cell profiling of COVID-19 patients in the recovery stage by single-cell sequencing. Cell Discov..

[88] Wilk AJ (2020). A single-cell atlas of the peripheral immune response in patients with severe COVID-19. Nat. Med..

[89] Xiong Y (2020). Transcriptomic characteristics of bronchoalveolar lavage fluid and peripheral blood mononuclear cells in COVID-19 patients. Emerg. Microbes Infect..

[90] Zhu, J. et al. An integrative genomics approach to the reconstruction of gene networks in segregating populations. *Cytogenet. Genome Res*. **105**, 363–74, (2004). 10.1159/000078209 15237224

[91] Zhu J (2007). Increasing the power to detect causal associations by combining genotypic and expression data in segregating populations. PLoS Comput. Biol..

[92] Zhu, J. et al. Integrating large-scale functional genomic data to dissect the complexity of yeast regulatory networks. *Nat. Genet*. **40**, 854–61, (2008). 10.1038/ng.167 PMC2573859 18552845

