# Supplementary material for: Downregulation of exhausted cytotoxic T cells in gene expression networks of multisystem inflammatory syndrome in children
Source: Nat Commun. 2021 Aug 11;12:4854. doi: 10.1038/s41467-021-24981-1 (PMC8357784; doi:10.1038/s41467-021-24981-1)
Supplement: Supplementary file 1 — Supplementary Information [file 41467_2021_24981_MOESM1_ESM.pdf]

Downregulation of exhausted cytotoxic T cells in gene expression networks of multisystem inflammatory syndrome in children

Noam D. Beckmann[1,2*], Phillip H. Comella[1,2,3*], Esther Cheng[1,3*], Lauren Lepow[4*], Aviva G. Beckmann[1], Scott R. Tyler[1], Konstantinos Mouskas[1], Nicole W. Simons[1], Gabriel E. Hoffman[1], Nancy J. Francoeur[1,2], Diane Marie Del Valle[5], Gurpawan Kang[6], Anh Do[1,2], Emily Moya[1], Lillian Wilkins[1], Jessica Le Berichel[5,], Christie Chang[7], Robert Marvin[7], Sharlene Calorossi[7], Alona Lansky[7], Laura Walker[7], Nancy Yi[7], Alex Yu[1], Jonathan Chung[5], Matthew Hartnett[8], Melody Eaton[7], Sandra Hatem[5], Hajra Jamal[9], Alara Akyatan[10], Alexandra Tabachnikova[9], Lora E. Liharska[1], Liam Cotter[1,3], Brian Fennessy[1], Akhil Vaid[1], Guillermo Barturen[11], Hardik Shah[1], Ying-chih Wang[1], Shwetha Hara Sridhar[1], Juan Soto[1,2], Swaroop Bose[1,2], Kent Madrid[1,2], Ethan Ellis[1,2], Elyze Merzier[1,2], Konstantinos Vlachos[1,2], Nataly Fishman[1,2], Manying Tin[1,2], Melissa Smith[1,2], Hui Xie[7,9], Manishkumar Patel[7,9], Kai Nie[7,9], Kimberly Argueta[7,9], Jocelyn Harris[7,9], Neha Karekar[7,9], Craig Batchelor[7,9], Jose Lacunza[7,9], Mahlet Yishak[7,9], Kevin Tuballes[7,9], Ieisha Scott[7,9], Arvind Kumar[3], Suraj Jaladanki[1], Charuta Agashe[7,9], Ryan Thompson[1,2], Evan Clark[1], Bojan Losic[1], Lauren Peters[1], The Mount Sinai COVID-19 Biobank Team[+], Panagiotis Roussos[1,2,4], Jun Zhu[8], Wenhui Wang[8], Andrew Kasarskis[8], Benjamin S. Glicksberg[1], Girish Nadkarni[12,13,14,15], Dusan Bogunovic[1], Cordelia Elaiho[16], Sandeep Gangadharan[17], George Ofori-Amanfo[17], Kasey Alesso-Carra[17], Kenan Onel[1,17], Karen M. Wilson[17], Carmen Argmann[1], Supinda Bunyavanich[1,2,17], Marta E. Alarcón-Riquelme[1], Thomas U. Marron[5,7], Adeeb Rahman[5,7,9,18], Seunghee Kim-Schulze[5,7,9,18], Sacha Gnjatic[5,7,9,18,19,20&], Bruce D. Gelb[1,17,21&], Miriam Merad[5,7,9,18&], Robert Sebra[1,2,22,23&], Eric E. Schadt[1,2,23,&], Alexander W. Charney[1,2,4,12&]

[*] Equally contributing authors
[&] Jointly supervising authors.
[+]*A list of authors and their affiliations appears at the end of the paper.*

Corresponding authors: Noam D. Beckmann (noam.beckmann@mssm.edu), Eric E. Schadt (eric.schadt@mssm.edu), Alexander W. Charney (alexander.charney@mssm.edu)

Supplementary Figures and Data

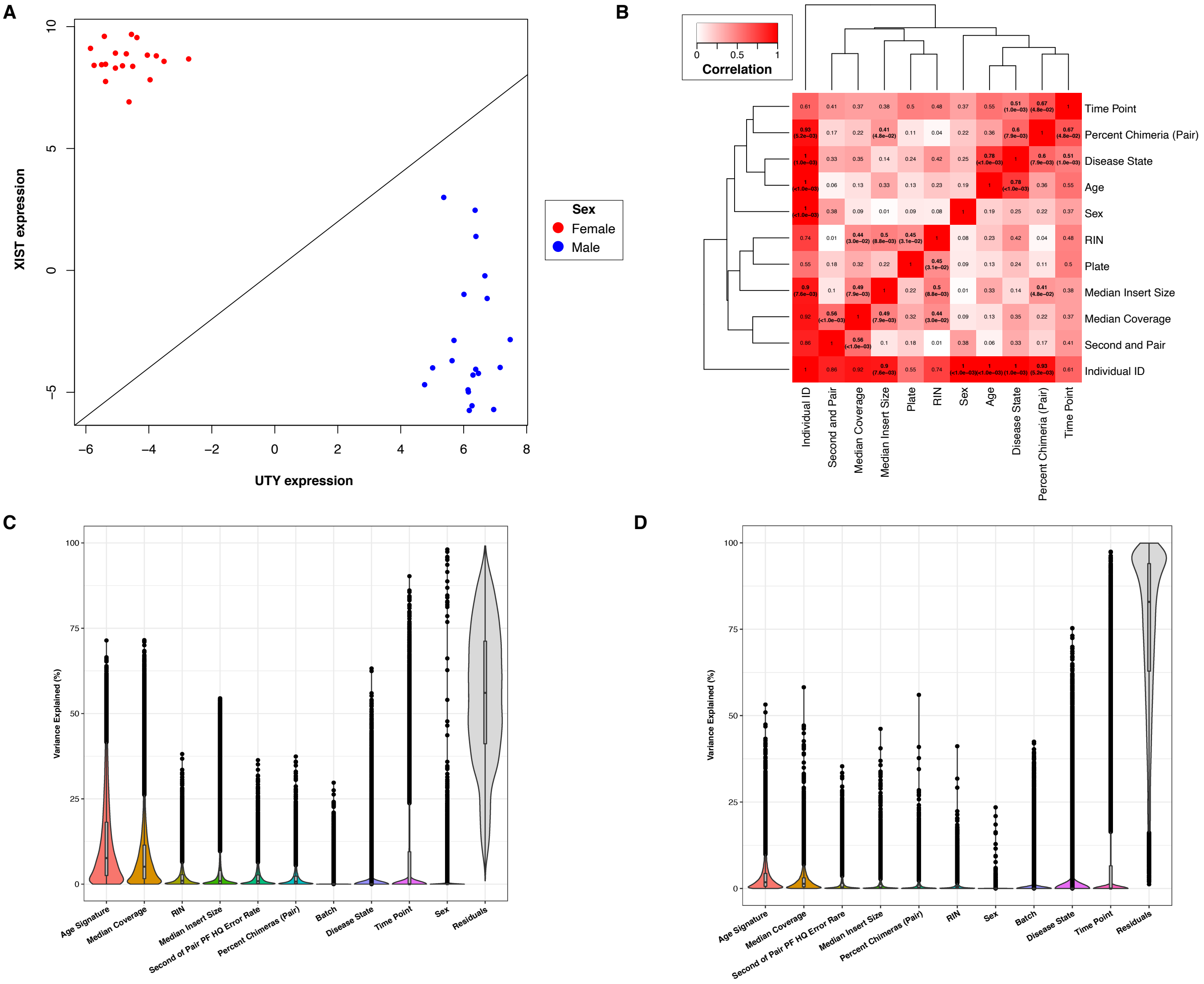

Supplementary Fig. 1: Quality control

A: Scatter plot of normalized expression of sex specific genes show no clear mislabeling. The x-axis is the UTY gene expression (Y chromosome gene) and the y-axis is the XIST gene. The color of the dots represents the sex as reported in the electronic medical records and is defined in the legend. The 1:1 line was added for ease of reading. **B:** Canonical correlation heatmap of important technical and biological covariates. The heat represents the correlation as defined in the legend and the correlation is shown in each box. The FDR adjusted permutation P-values are shown for significant correlation (Permutation testing). **C, D:** Variance partition violin plot before **C:** and after **D:** covariate adjustment. X-axes are the different covariates and the y-axes are percentages of variation explained by each covariate for each gene.

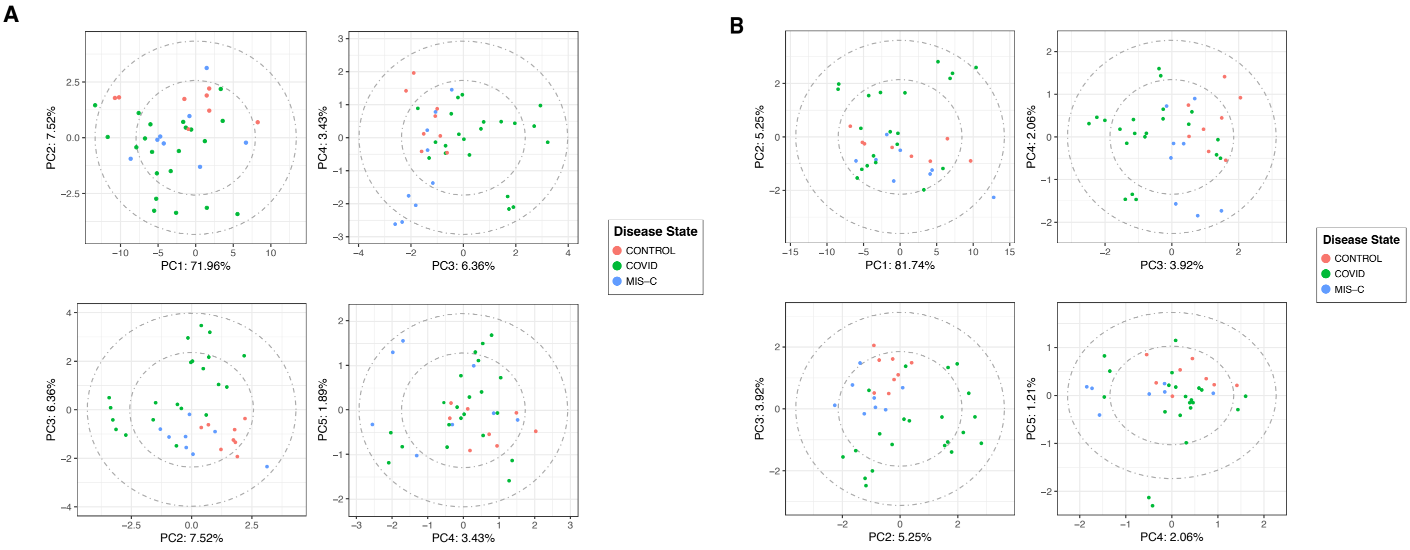

Supplementary Fig. 2: Principal component analyses before (A) and after (B) adjustment for imputed age.

A, B: X and y-axes are the principle components and the amount of variation explained by them. Points are colored by disease states.

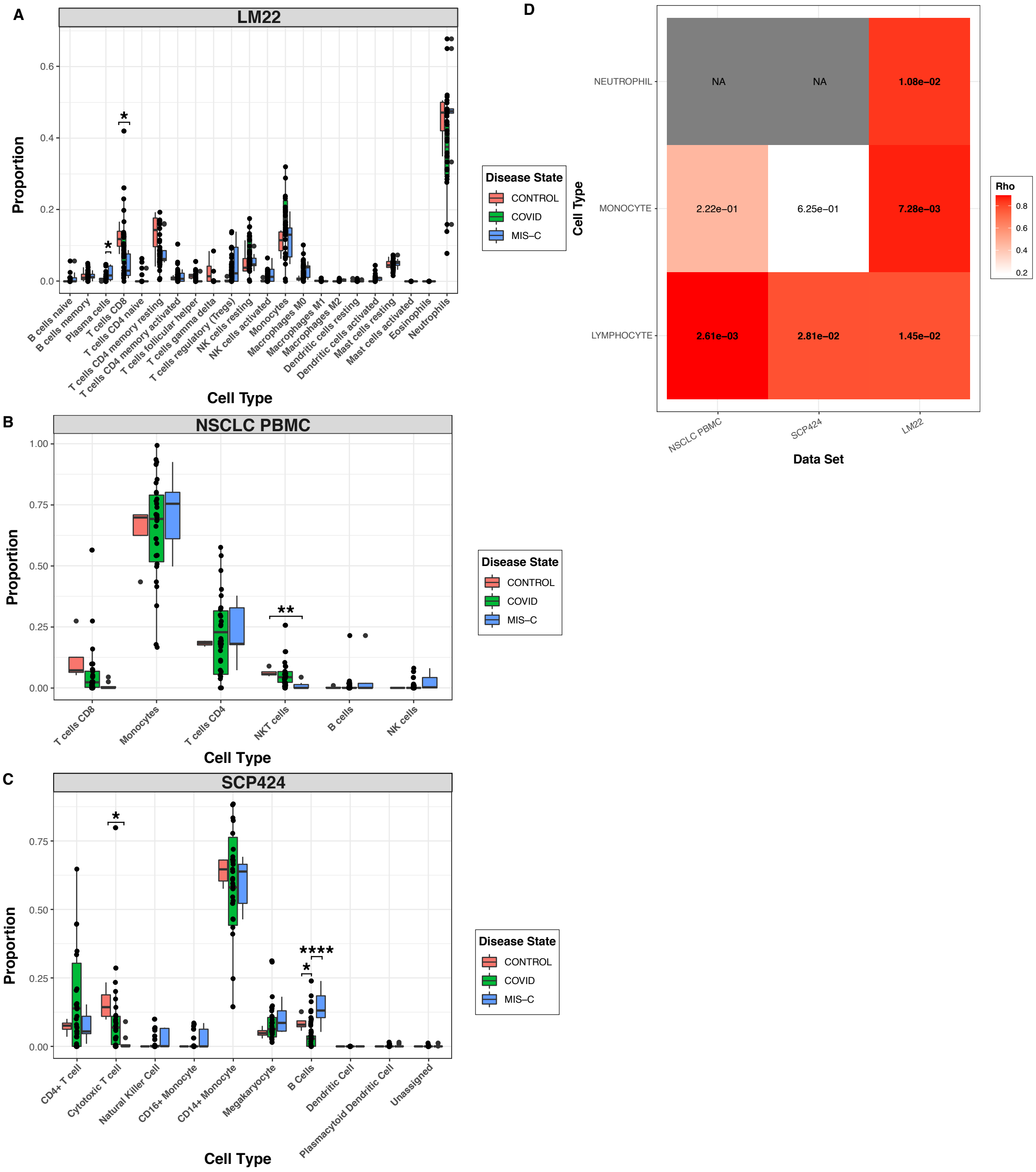

Supplementary Fig. 3: Cell type deconvolution results.

A, B, C: Box plots showing cell type deconvolution using three references (performed using a linear model). The x-axes represent deconvoluted cell type as defined in the references, where **A:** and **B:** are from Newman, et al, Nature Biotech, 2019, and **C:** is from Ding, et al, BioRxiv, May 2019. The y-axes are the proportion of the deconvolved expression attributed to each cell type.

The color of the box plot represents the disease state as defined in the legend. All p-values were adjusted for multi-testing as described in *Methods*. **D:** The correlation heatmap of deconvoluted cell type and measured cell blood count (CBC). The x-axis represents the deconvolution references used for deconvolution, as stated above. The y-axis is the CBC measurements. The color of the heat represents the correlation (performed using R two sided Pearson Correlation test) and adjusted p-values (as defined in *Methods*) are shown on the plot. Significant adjusted p-values are shown in bold.

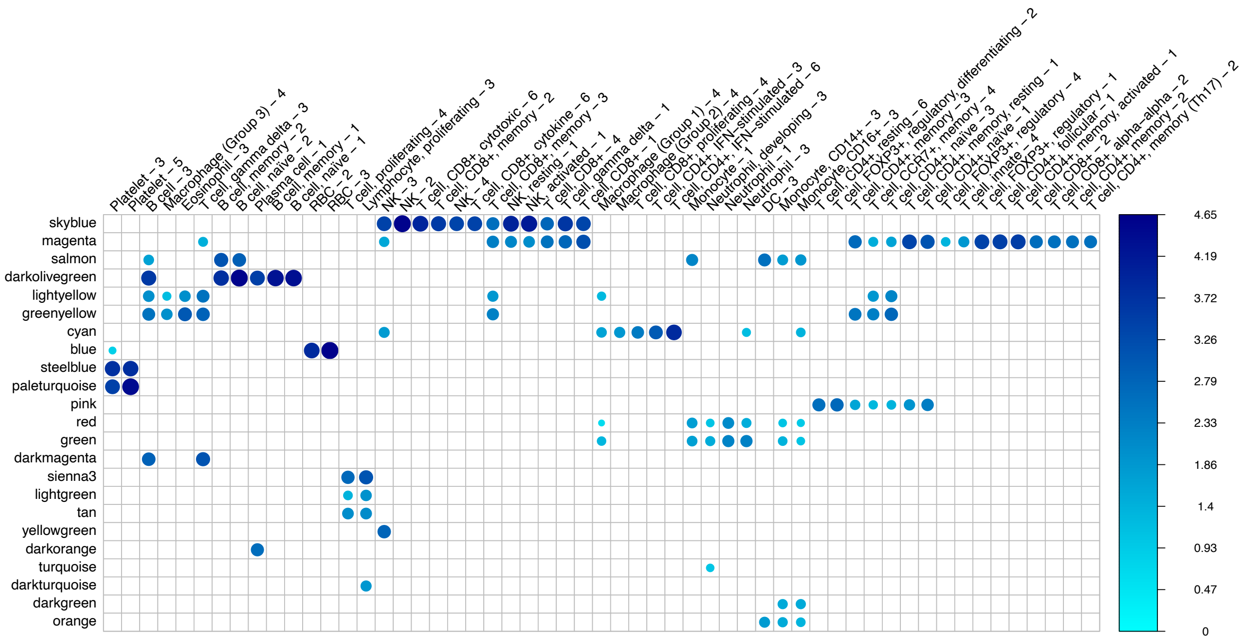

Supplementary Fig. 4: Module enrichments for cell-type specific signatures.
The x-axis are the modules and the y-axis the cell-types and reference indices: 1= Newman et al, Nature Methods, 2015, 2= Park, et al, Science, 2020, 3= Wilk, et al, Nature Medicine, 2020, 4= Lial, Nature Medicine, 2020, 5= Rowley, et al, Blood, 2011, and 6= Szabo, Nature Communications, 2019. The color and size of the circles represent the log(odds ratio) for the enrichment of the modules for the cell type specific signatures as defined in the legend (performed using Fisher's test).

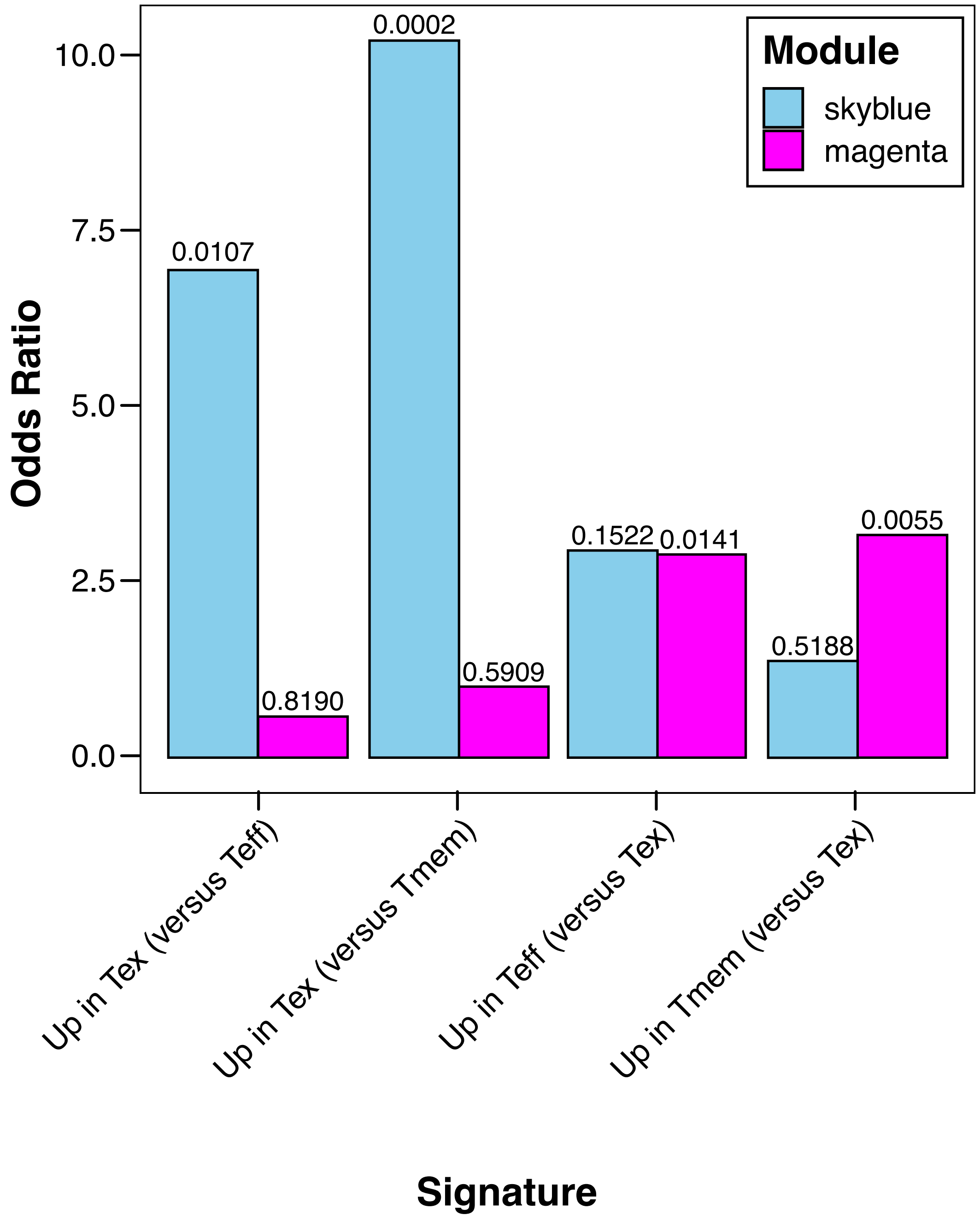

Supplementary Fig. 5: Projection of $CD8^+$ Tcell subtypes signatures in skyblue and magenta modules.

The x-axis is the name of the signature projected onto the modules as defined in Wherry et al, Immunity, 2007, and the y-axis is the OR for the enrichment of the corresponding signature in the module. The colors of the modules are representative of the module names and are defined in the legend. Enrichment p-values are shown above each bar (performed using Fisher's test). All p-values were adjusted for multi-testing as described in *Methods*.

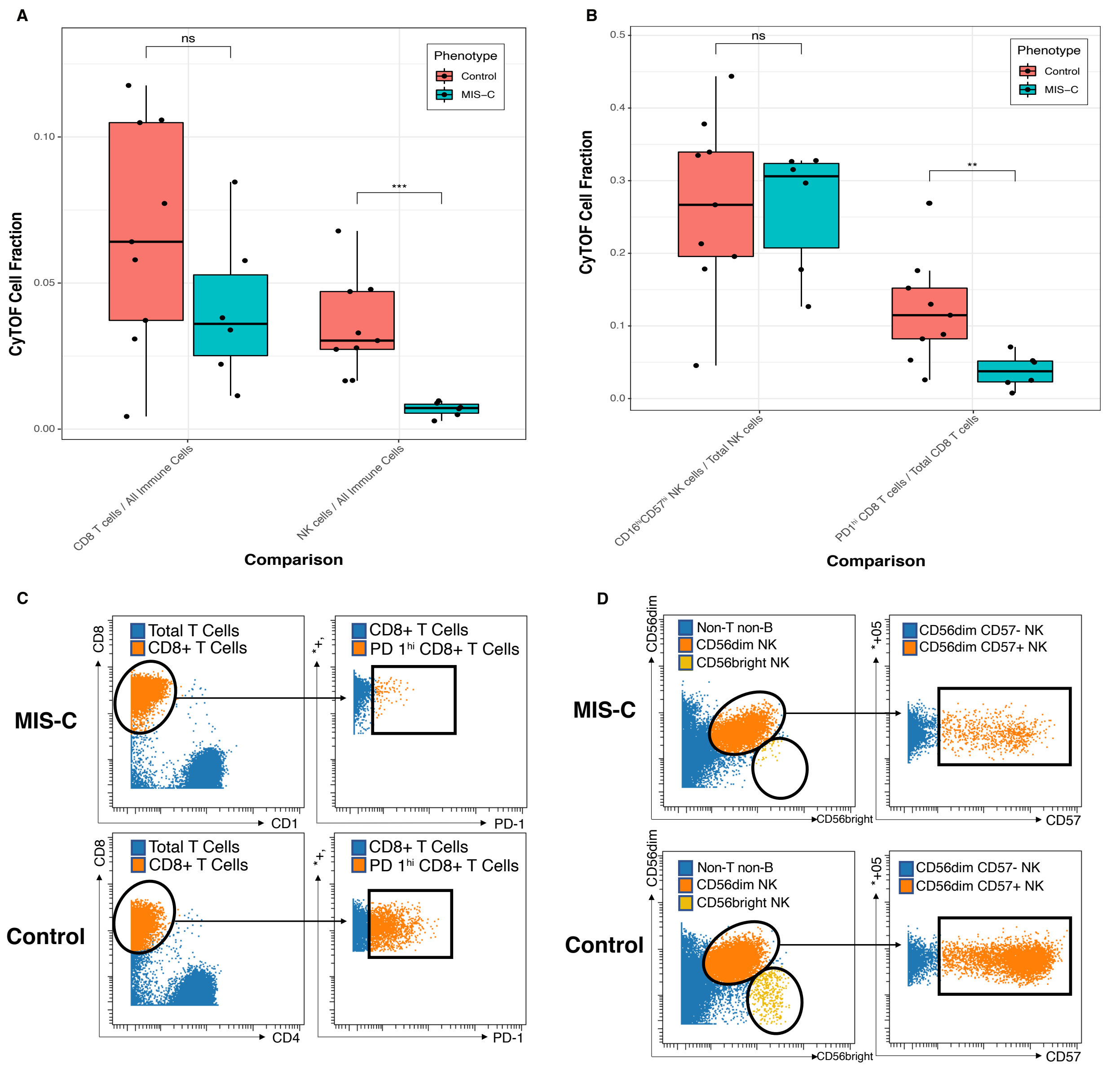

Supplementary Fig. 6: CyTOF validation.

A, B: Box plots of cell fractions quantified with CyTOF between MIS-C and HCs for **A**: $CD8^{+}$ Tcells and NK cells and **B:** $CD56^{dim}CD57^{+}$ NK cells and PD-1^{high} $CD8^{+}$ Tcells. The x-axes represent the cell type assessed and the denominator cell population and the y axes are the fractions of cells belonging to the cell types assessed (performed using one sided Wilcoxon signed-rank test). Significant adjusted p- values were respectively **A:** p = 0.0004 and **B:** p = 0.00480. The color of the box plot represents the disease state as defined in the legend. **C, D:** Representative biaxial plots for **C:** PD-1^{high} $CD8^{+}$ T cells and **D:** $CD56^{dim}CD57^{+}$ NK cells. Each figure consists of 2 rows, with a MIS-C representative sample on top and a HC representative sample on the bottom, as

well as 2 columns to define the population of cells of interest. For each panel, the x and y axes contain a logarithmic scale of cell counts for cell surface markers defined on the axes. The colors of the points in the panels are defined in the panel legends. All p-values were adjusted for multi-testing as described in *Methods*.
