# Supplementary material for: Downregulation of exhausted cytotoxic T cells in gene expression networks of multisystem inflammatory syndrome in children
Source: Nat Commun. 2021 Aug 11;12:4854. doi: 10.1038/s41467-021-24981-1 (PMC8357784; doi:10.1038/s41467-021-24981-1)
Supplement: Supplementary file 2 — Reporting Summary [file 41467_2021_24981_MOESM2_ESM.pdf]

nature research

Corresponding author(s): Alexander Charney
Noam Beckmann
Eric Schadt

Last updated by author(s): 4/29/2021

Reporting Summary

Nature Research wishes to improve the reproducibility of the work that we publish. This form provides structure for consistency and transparency in reporting. For further information on Nature Research policies, see our Editorial Policies and the Editorial Policy Checklist.

Statistics

For all statistical analyses, confirm that the following items are present in the figure legend, table legend, main text, or Methods section.

n/a	Confirmed	
☐	☒	The exact sample size (*n*) for each experimental group/condition, given as a discrete number and unit of measurement
☐	☒	A statement on whether measurements were taken from distinct samples or whether the same sample was measured repeatedly
☐	☒	The statistical test(s) used AND whether they are one- or two-sided<br>*Only common tests should be described solely by name; describe more complex techniques in the Methods section.*
☐	☒	A description of all covariates tested
☐	☒	A description of any assumptions or corrections, such as tests of normality and adjustment for multiple comparisons
☐	☒	A full description of the statistical parameters including central tendency (e.g. means) or other basic estimates (e.g. regression coefficient) AND variation (e.g. standard deviation) or associated estimates of uncertainty (e.g. confidence intervals)
☐	☒	For null hypothesis testing, the test statistic (e.g. *F*, *t*, *r*) with confidence intervals, effect sizes, degrees of freedom and *P* value noted<br>*Give P values as exact values whenever suitable.*
☐	☒	For Bayesian analysis, information on the choice of priors and Markov chain Monte Carlo settings
☐	☒	For hierarchical and complex designs, identification of the appropriate level for tests and full reporting of outcomes
☐	☒	Estimates of effect sizes (e.g. Cohen's *d*, Pearson's *r*), indicating how they were calculated

Our web collection on statistics for biologists contains articles on many of the points above.

Software and code

Policy information about availability of computer code

Data collection	No code was used for data collection.
Data analysis	bcl2fastq Conversion tool (Illumina)<br>STAR (v2.7.3a)<br>fastqc (v0.11.8)<br>Picard Tools (v2.22.3)<br>kallisto (v0.46.1)<br>Subread R package (v1.6.3)<br>MultiQC (v1.9.dev0)<br>NGSCheckMate (v1.0.0)<br>limma R package (v3.44.3)<br>variancePartition R package (v1.19.6)<br>CCP R package (v1.1)<br>CIBERSORTx (v1.0)<br>goseq R package (v1.40.0)<br>topGO R package (v2.40.0)<br>org.Hs.eg.db R package (v3.11.4)<br>HTSanalyzeR R package (v2.3.5)<br>GSEABase R package (1.50.1)<br>GAGE R package (v2.38.3)<br>WGCNA R package (v1.69)<br>GEOquery R package (v2.56.0)

illuminaHumanv4.db R package (v1.26.0)
HUGO (website)
biomaRt R package (v2.44.1)
RIMBAnet software (v1.0)
Key Driver Analysis R package (v1.0)
Fluidigm (v6.7.1016)

For manuscripts utilizing custom algorithms or software that are central to the research but not yet described in published literature, software must be made available to editors and reviewers. We strongly encourage code deposition in a community repository (e.g. GitHub). See the Nature Research guidelines for submitting code & software for further information.

Data

Policy information about availability of data

All manuscripts must include a data availability statement. This statement should provide the following information, where applicable:

- Accession codes, unique identifiers, or web links for publicly available datasets
- A list of figures that have associated raw data
- A description of any restrictions on data availability

The datasets generated during and/or analyzed during the current study are available on Sage Synapse: syn25590355.

Field-specific reporting

Please select the one below that is the best fit for your research. If you are not sure, read the appropriate sections before making your selection.

- [x] Life sciences
- [ ] Behavioural & social sciences
- [ ] Ecological, evolutionary & environmental sciences

For a reference copy of the document with all sections, see nature.com/documents/nr-reporting-summary-flat.pdf

Life sciences study design

All studies must disclose on these points even when the disclosure is negative.

Sample size	MIS-C cases (N=8), Pediatric COVID-19 cases (N = 7), Healthy Controls (N = 4). This is a molecule study of a new rare disease, sample size was determined based on the prevalence of the condition in our hospital system.
Data exclusions	All MIS-C cases met the formal MIS-C diagnostic criteria and had positive SARS-CoV-2 serology at the time of sampling. Data was only excluded based on sequencing quality metrics.
Replication	Replication was addressed when analyzing co-expression module enrichment by using many external datasets to show replication of disease signature enrichment signal. Replication was also addressed when analyzing cell type deconvolution by using 3 separate references.
Randomization	Recruitment of age-matched healthy controls was not feasible during the study period due to the risk of exposing healthy children to COVID-19 by coming to the hospital for a research blood draw. Age effect was modeled by deriving an estimate of the transcriptional age of each study participant. This approach allowed us to remove the effect of age on gene expression without removing the effects of disease, as would happen if age itself were included as a covariate in our analyses
Blinding	The was not a randomized control trial, patients were recruited as they presented to the hospital. Blinding in the context of this study design is to our knowledge, not something that is done. Blinding at the point of sample collection is not possible, blinding at the stage of sample prep and data generation due to the need for proper randomization of cases and controls across batches. Blinding would decrease the integrity of the science in this context.

Reporting for specific materials, systems and methods

We require information from authors about some types of materials, experimental systems and methods used in many studies. Here, indicate whether each material, system or method listed is relevant to your study. If you are not sure if a list item applies to your research, read the appropriate section before selecting a response.

Materials & experimental systems

n/a	Involved in the study	
x		Antibodies
x		Eukaryotic cell lines
x		Palaeontology and archaeology
x		Animals and other organisms
	x	Human research participants
x		Clinical data
x		Dual use research of concern

Methods

n/a	Involved in the study	
x		ChIP-seq
x		Flow cytometry
x		MRI-based neuroimaging

Human research participants

Policy information about studies involving human research participants

Population characteristics	Data covariates can be found in Table 1 and Supplementary Table 1. MIS-C cases (N=8) ranged in age from five to 20 years old (mean = 11, standard deviation = 5.2), were largely free of other comorbidities, and balanced for gender. Pediatric COVID-19 cases (N = 7) ranged in age between 5 months and 19 years old (mean age = 12.4, standard deviation = 7). Nearly all of pediatric COVID-19 cases were chronically immunocompromised, with three suffering from comorbid malignancies requiring chemotherapy and three on immunosuppressive therapy for other indications. Healthy Controls (HCs) ranged in age from 24 to 37 (N = 4, mean age = 30.75, standard deviation = 6.18), were free of other comorbidities, and 3 of 4 were female.
Recruitment	MIS-C cases and Pediatric COVID-19 cases were recruited as the presented in the hospital during the pandemic. Health Controls were recruited from research personnel working at the hospital working during the pandemic. This generated a bias in age with Healthy Controls being older. This covariate was adjusted for per the methods section.
Ethics oversight	Icahn School of Medicine at Mount Sinai's Institutional Review Board

Note that full information on the approval of the study protocol must also be provided in the manuscript.
