# Supplementary material for: Downregulation of exhausted cytotoxic T cells in gene expression networks of multisystem inflammatory syndrome in children
Source: Nat Commun. 2021 Aug 11;12:4854. doi: 10.1038/s41467-021-24981-1 (PMC8357784; doi:10.1038/s41467-021-24981-1)
Supplement: Supplementary file 3 — Description of Additional Supplementary Files [file 41467_2021_24981_MOESM3_ESM.pdf]

Description of Additional Supplementary Files

File Name: SupplementaryData01_Treatments
Description: Table of treatments per patient at time of RNA-seq.

File Name: SupplementaryData02_MISCSymptoms
Description: Table of MIS-C symptoms per each patient at time of RNA-seq.

File Name: SupplementaryData03_Timeline
Description: Patient care timelines.

File Name: SupplementaryData04_AgeSignatureGenes
Description: List of all genes that were used to create an age adjustment via principal component analysis of our dataset.

File Name: SupplementaryData05_CelltypeDeconvolution
Description: Table using the output of the cell type deconvolution analysis to compare differing proportions of cell types between our samples.

File Name: SupplementaryData06_DeconvolutionCorrelationToCBC
Description: Correlation of the cell type deconvolution analysis and the complete blood count of patients.

File Name: SupplementaryData07_DifferentialExpressionTable
Description: Output of differential expression analysis between COVID19, MIS-C, and HC samples.

File Name: SupplementaryData08_DESignatureGOEnrichment
Description: Enrichment table of GO terms and the differential expression signatures generated from sheet 1DifferentialExpressionTable.

File Name: SupplementaryData09_CellTypeSignatureEnrichments
Description: Enrichment table of published cell type signatures and their enrichments with our coexpression modules.

File Name: SupplementaryData10_GOTermEnrichment
Description: Enrichment table of GO terms and our coexpression modules.

File Name: SupplementaryData11_MSigDBC7TermEnrichment
Description: Enrichment table of MSigDB "C7" terms and our coexpression modules.

File Name: SupplementaryData12_MSigDBHallmarkTermEnrichment
Description: Enrichment table of MSigDB "Hallmark" terms and our coexpression modules.

File Name: SupplementaryData13_AgeMatchedMISCEnrichments
Description: Validation table of coexpression module enrichments for signature from age matched controls.

File Name: SupplementaryData14_DiffExpressionAgeMatch

Description: Differential expression signatures between this study's healthy controls and an external age matched healthy control validation group.

File Name: SupplementaryData15_DiseaseSignatureEnrichments
Description: Enrichment table of published disease signatures and their enrichments with our coexpression modules.

File Name: SupplementaryData16_SkyblueCelltypeDissection
Description: Enrichment table of already published cell type signatures specific for NK - T cell subsets and their enrichments with our skyblue module.

File Name: SupplementaryData17_SkyblueNKTcellEnrichment
Description: Enrichment table for skyblue module and NK and CD8+ T cell subtypes.

File Name: SupplementaryData18_CyTOFCellFraction
Description: Table of CyTOF cell fractions.

File Name: SupplementaryData19_CyTOFAbInfo
Description: Table for antibody information used in the CyTOF experiment.

File Name: SupplementaryData20_ModuleKeyDrivers
Description: Output of Key Driver Analysis using the Bayesian Network for our co-expression modules.

File Name: SupplementaryData21_CoexpressionNetwork
Description: Output of the coexpression analysis. List of genes in our dataset and their module membership.
